# Supplementary material for: Using set visualisation to find and explain patterns of missing values: a case study with NHS hospital episode statistics data
Source: BMJ Open. 2022 Nov 21;12(11):e064887. doi: 10.1136/bmjopen-2022-064887 (PMC9680176; doi:10.1136/bmjopen-2022-064887)
Supplement: Author's manuscript [file bmjopen-2022-064887.draft_revisions.pdf]

BMJ Open is committed to open peer review. As part of this commitment we make the peer review history of every article we publish publicly available.

When an article is published we post the peer reviewers' comments and the authors' responses online. We also post the versions of the paper that were used during peer review. These are the versions that the peer review comments apply to.

The versions of the paper that follow are the versions that were submitted during the peer review process. They are not the versions of record or the final published versions. They should not be cited or distributed as the published version of this manuscript.

BMJ Open is an open access journal and the full, final, typeset and author-corrected version of record of the manuscript is available on our site with no access controls, subscription charges or pay-per-view fees (http://bmjopen.bmj.com).

If you have any questions on BMJ Open's open peer review process please email info.bmjopen@bmj.com

BMJ Open

Using set visualization techniques to investigate and explain patterns of missing values in electronic health records

Journal:	*BMJ Open*
Manuscript ID	bmjopen-2022-064887
Article Type:	Original research
Date Submitted by the Author:	20-May-2022
Complete List of Authors:	Ruddle, Roy; University of Leeds, School of Computing and Leeds Institute for Data Analytics<br>Adnan, Muhammad; Higher Colleges of Technology, Computer Science<br>Hall, Marlous ; University of Leeds, Institute of Cardiovascular & Metabolic Medicine and Leeds Institute for Data Analytics
Keywords:	Health informatics < BIOTECHNOLOGY & BIOINFORMATICS, STATISTICS & RESEARCH METHODS, QUALITATIVE RESEARCH

SCHOLARONE™
Manuscripts

BMJ

I, the Submitting Author has the right to grant and does grant on behalf of all authors of the Work (as defined in the below author licence), an exclusive licence and/or a non-exclusive licence for contributions from authors who are: i) UK Crown employees; ii) where BMJ has agreed a CC-BY licence shall apply, and/or iii) in accordance with the terms applicable for US Federal Government officers or employees acting as part of their official duties; on a worldwide, perpetual, irrevocable, royalty-free basis to BMJ Publishing Group Ltd ("BMJ") its licensees and where the relevant Journal is co-owned by BMJ to the co-owners of the Journal, to publish the Work in this journal and any other BMJ products and to exploit all rights, as set out in our licence.

The Submitting Author accepts and understands that any supply made under these terms is made by BMJ to the Submitting Author unless you are acting as an employee on behalf of your employer or a postgraduate student of an affiliated institution which is paying any applicable article publishing charge ("APC") for Open Access articles. Where the Submitting Author wishes to make the Work available on an Open Access basis (and intends to pay the relevant APC), the terms of reuse of such Open Access shall be governed by a Creative Commons licence – details of these licences and which Creative Commons licence will apply to this Work are set out in our licence referred to above.

Other than as permitted in any relevant BMJ Author's Self Archiving Policies, I confirm this Work has not been accepted for publication elsewhere, is not being considered for publication elsewhere and does not duplicate material already published. I confirm all authors consent to publication of this Work and authorise the granting of this licence.

For peer review only

Title: Using set visualization techniques to investigate and explain patterns of missing values in electronic health records

Authors

Corresponding author: Roy A. Ruddle, School of Computing and Leeds Institute for Data Analytics, University of Leeds, Leeds, LS2 9NL, UK, r.a.ruddle@leeds.ac.uk

Muhammad Adnan, Higher Colleges of Technology, University City, Sharjah, UAE, madnan1@hct.ac.ae

Marlous Hall, Institute of Cardiovascular & Metabolic Medicine and Leeds Institute for Data Analytics, University of Leeds, Leeds, UK, m.s.hall@leeds.ac.uk

For peer review only

For peer review only - http://bmjopen.bmj.com/site/about/guidelines.xhtml

I, the Submitting Author has the right to grant and does grant on behalf of all authors of the Work (as defined in the below author licence), an exclusive licence and/or a non-exclusive licence for contributions from authors who are: i) UK Crown employees; ii) where BMJ has agreed a CC-BY licence shall apply, and/or iii) in accordance with the terms applicable for US Federal Government officers or employees acting as part of their official duties; on a worldwide, perpetual, irrevocable, royalty-free basis to BMJ Publishing Group Ltd ("BMJ") its licensees and where the relevant Journal is co-owned by BMJ to the co-owners of the Journal, to publish the Work in BMJ Open and any other BMJ products and to exploit all rights, as set out in our licence.

The Submitting Author accepts and understands that any supply made under these terms is made by BMJ to the Submitting Author unless you are acting as an employee on behalf of your employer or a postgraduate student of an affiliated institution which is paying any applicable article publishing charge ("APC") for Open Access articles. Where the Submitting Author wishes to make the Work available on an Open Access basis (and intends to pay the relevant APC), the terms of reuse of such Open Access shall be governed by a Creative Commons licence – details of these licences and which Creative Commons licence will apply to this Work are set out in our licence referred to above.

For peer review only

ABSTRACT

Objectives: Missing data is the most common data quality issue in electronic health records (EHRs). Checks are typically limited to counting the number of missing values in individual fields, but researchers and organisations need to understand multi-field missing data patterns, and counts or numerical summaries are poorly suited to that. This study shows how set-based visualization enables multi-field missing data patterns to be discovered and investigated.

Design: Development and evaluation of interactive set visualization techniques to find patterns of missing data and generate actionable insights.

Setting and participants: Anonymised Admitted Patient Care health records for NHS hospitals and independent sector providers in England. The visualization and data mining software was run over 16 million records and 86 fields in the dataset.

Results: The dataset contained 960 million missing values. Set visualization bar charts showed how those values were distributed across the fields, including several fields that, unexpectedly, were not complete. Set intersection heatmaps revealed unexpected gaps in diagnosis, operation and date fields. Information gain ratio and entropy calculations allowed us to identify the origin of each unexpected pattern, in terms of the values of other fields.

Conclusions: Our findings show how set visualization reveals important insights about multi-field missing data patterns in large EHR datasets. The study revealed both rare and widespread data quality issues that were previously unknown to an epidemiologist, and allowed a particular part of a specific hospital to be pinpointed as the origin of rare issues that NHS Digital did not know exist.

Keywords: Data visualization; data quality; missing values; electronic health records

Word count: 4,015

For peer review only - http://bmjopen.bmj.com/site/about/guidelines.xhtml

ARTICLE SUMMARY

Strengths and limitations of this study

- This study demonstrates the utility of interactive set visualization techniques for finding and explaining patterns of missing values in electronic health records, irrespective of whether those patterns are common or rare.
- The techniques were evaluated in a case study with a large (16-million record; 86 field) Admitted Patient Care dataset from NHS hospitals.
- There was only one data table in the dataset. However, ways to adapt the techniques for longitudinal data and relational databases are described.
- The evaluation only involved one dataset, but that was from a national organisation that provides many similar datasets each year to researchers and organisations.

INTRODUCTION

Missing data occurs more often in electronic health records (EHRs) than any other single data quality issue.[1] The motivating problem for the present research was how to investigate patterns of missing data that involve many variables and explain those patterns by identifying the underlying structures.[2] Our objective was to develop and evaluate a method to achieve that using set visualization, to enable users to generate actionable insights from datasets that contain millions of records. To be successful, the method needs to reveal both known and unknown patterns, including those that are often obscured in a dataset due to their rarity. To help ensure that the visualization method was scalable we chose a set-based approach, but limited the scope of the research to analysing flat file data tables. The research used the TRIPOD reporting guidelines.[3]

Researchers need to investigate patterns of missing values to understand possible effects on cohort selection, bias, impact on data linkages and to design appropriate missing data techniques such as multiple imputation.[1 4-8] Organisations collecting and providing such data strive to continuously improve data quality.[9-12] We collaborate with one such organisation, NHS Digital, which is the UKs national information and technology service collecting more than 100 million records/year of Hospital Episode Statistics (HES) [13] for the National Health Service in England. HES data are directly used in the payment

For peer review only - http://bmjopen.bmj.com/site/about/guidelines.xhtml

of hospitals throughout England, as well as for secondary uses such as research, quality indicators and planning health services.[8 14]

At present, the only checks that researchers and organisations typically perform are basic counts of missing values in individual fields.[6 7 9 12 15-17] In NHS Digital's case those checks are limited to core data fields, and if the percentage missing exceeds a threshold (30%) then feedback is sent to the hospital.[10 12] However, researchers also want to investigate patterns of missing values that involve multiple fields,[1 18] and NHS Digital wants to do that to define new business rules for data cleaning and to cross-reference variations in data quality with external influences (e.g. changes in policy or coding improvement initiatives).

Patterns of missing values may be computed and/or visualized.[2] Some computations model the distribution of missing values to estimate whether they adhere to a predefined pattern and to allow the analysts to consider assumptions about missing at random (MAR), missing completely at random (MCAR) or missing not at random patterns (MNAR [19]), with the latter including monotone and unit non-response ("block") patterns.[20] However these methods rely on probabilistic models, so by their very nature, they are poorly suited for detecting rare patterns of missingness.

An alternative approach is to use set analysis. Imagine a dataset has three fields (A, B and C). Set A is all of the records that are missing the value for Field A, and similarly for sets B and C. There are four possible exclusive set intersections (AB, AC, BC and ABC), where intersection AB contains the records that are only missing values for Fields A and B, and so on. The number of intersections cannot be greater than the number of records in the dataset,[21] so it is computationally tractable to identify every combination of fields that are missing together irrespective of whether it occurs millions of time or only once.

Sophisticated visualization tools and techniques have been developed for cohort selection, risk analysis and other types of detailed EHR data analysis,[22-24] but data quality use cases are a notable omission [25]. Visualization dashboards are widely used in healthcare,[26] but focus on high-level metrics such as the number of values that are missing from key fields, which corresponds to visualizing specific sets of missing data. However, it is not sufficient for tools to visualize sets of missing data because users need to visualize set intersections if they are to investigate multi-field patterns of missing values in detail. Most tools to date adopt designs that are unsuitable for showing set intersections, [27-29] and other only

For peer review only - http://bmjopen.bmj.com/site/about/guidelines.xhtml

display pairwise intersections,[30 31] or require excessive interaction because users have to select specific fields of interest.[32-34]

The most appropriate technique for visualizing set intersections is a matrix of sets vs. intersections. That technique is provided by VIM,[35] but only as static plots. UpSet [36] provides interactive set intersection visualization but, when the present research was conducted, the latest version (UpSet2) only allowed 5 MB of data to be loaded and our dataset was one thousand times larger. Since then, more scalable R and Python UpSet implementations have been released [37 38] but, like VIM they also generate static plots.

MATERIALS AND METHODS

Setting and dataset

We used a 15,733,889 record extract of Admitted Patient Care (APC) data from 2016-17 hospital episode statistics (HES) from NHS Digital. APC data contains information about admissions to NHS hospitals in England and to independent sector providers paid for by the NHS, and is collected for clinical uses, determining payments to hospitals, care quality reports and a wide variety of research [16]. Our extract was from a project that was studying long-term outcomes and hospitalisation rates for acute myocardial infarction (heart attack) patients, and requested help investigating the quality of their data. The cohort comprised patients who were aged 18 or older at 31st March 2009, and had not had an acute myocardial infarction during years 2001/02 to 2007/08.

The dataset was processed to flag any “unknown” values in each field [12] as missing, and had 86 fields that may be divided into four groups. Twenty fields contained ICD-10 diagnosis codes for a patient's illness or condition (DIAG_01 to DIAG_20), which should be filled in incrementally and exhibit a monotone pattern of missing data. Similarly, 24 fields contained codes about a patient's operations (OPERTN_01 to OPERTN_24) and 24 fields contained the corresponding dates for those operations (MYOPDATE_01 to MYOPDATE_24), with the operations and dates fields exhibiting monotone patterns, and each pair of operation/date fields exhibiting a block missingness pattern. There were 18 general fields: patient, episode and A&E record identifiers (ENCRYPTED_HESID; EPIKEY; AEKEY), episode order, status and type (EPIORDER; EPISTAT; EPITYPE), flags to indicate a finished admission and consultant episode (FAE; FCE), dates of admission, episode start and episode end (MYADMIDATE; MYEPISTART; MYEPIEND), patient date of birth, age and sex (MYDOB; ADMIAGE;

For peer review only - http://bmjopen.bmj.com/site/about/guidelines.xhtml

SEX), whether patient was alive or dead and time from episode end date to death (Mortality; SURVIVALTIME), admission method (ADMIMETH) and the hospital's code (PROCODE).

Visualization design

We designed a novel tool called ACE (Analysis of Combinations of Events), which is freely available for download and use.[39] The tool reads data from a flat file format (a CSV or other text file), calculates the ESIs, and stores the results in a memory-resident HyperSQL database.[40] ACE is implemented in Java and R, with a JavaFX graphical user interface.

ACE combines set-based visualization and data mining methods in an integrated, interactive tool that enables users to investigate multi-field missing data patterns. Figure 1 shows a recommended workflow, which starts with computing the sets (one for each field) and set intersections (each combination of missing fields), and then visualizing the sets (the number of values missing from each field) and distributions of the set intersections (to investigate high-level patterns). The substance of the workflow follows, with two levels of iteration.

[Figure 1 should go approximately here]

The user interface is based on a tab pane layout that allows users to: (1) see each iterative step taken during the analysis, (2) undo steps by closing tabs, and (3) compare patterns of missing values in different subsets of the data by switching tabs. An alternative to tabs would have been to integrate all of the visualizations into a single window, but that would have reduced the screen space available for each visualization and limit the detail that users could see without scrolling or zooming.

Two of the visualizations are for sets and four visualizations are for set intersections. The primary set visualization is called a *value bar chart* (Figure 2), which shows the number of missing values in each field (this is standard functionality in other tools [30-37 41-43]). That visualization is complemented by a *value count histogram*, which calculates bins for the number of missing values, shows the number of fields in each bin, and lets our tool scale to datasets with an unlimited number of fields. Both of the visualizations allow users to sort the fields, and to select certain fields for the next step in the analysis.

[Figure 2 should go approximately here]

For peer review only - http://bmjopen.bmj.com/site/about/guidelines.xhtml

The primary set intersection visualization is called a *combination heatmap* (Figure 3), which shows fields (X axis) × set intersections (Y axis) and the number of records that are in each intersection (colour). The height of the heatmap depends on the number of intersections, which makes it suitable for large datasets. By contrast, a missingness map [44] displays a separate row for each record in a dataset, so the case study's 16 million record dataset would produce a map with 16 million rows, which is clearly not practical.

[Figure 3 should go approximately here]

The heatmap fields may be sorted alphabetically or into dataset order, the intersections may be sorted by degree (number of missing fields) and cardinality (number of records), and the heatmap cells may be clustered using two heuristic sorting methods from a recent state-of-the-art report.[45] The heatmap is complemented by a *combination count histogram* and a *combination length histogram* (Figure 4) that calculate bins for the number of records and fields, respectively, and show the number of intersections in each bin. This lets ACE scale to an unlimited number of set intersections. The sixth visualization (a *combination bar chart*) is for intermediate scalability because it shows the number of records in each intersection.

[Figure 4 should go approximately here]

The tool's data mining methods comprise information gain ratio (IGR) [46] and entropy calculations, which are key concepts in information theory,[47] By selecting one or more intersections, the IGR enables users to rank other fields in terms of their ability at explaining certain intersections. Entropy enables users to identify which values of a specific field are strongly associated with the intersections. IGR output was displayed in a bar chart in its own tab, and entropy output was displayed in a bar chart or table in its own tab.

All of the above visualizations and data mining techniques can be applied to either the whole dataset or subsets of it, chosen by selecting or filtering-out fields and/or combinations. This lets users iteratively analyse multiple patterns of missing values that coexist in the dataset.

For peer review only - http://bmjopen.bmj.com/site/about/guidelines.xhtml

Case study

The ACE software was evaluated in a case study with a senior epidemiologist who was studying long-term outcomes and hospitalisation rates for survivors of acute myocardial infarction. During the case study, the epidemiologist and one of the software's developers worked together, with the latter controlling the user interface. This mimics the collaborative working environment that is found in many data science projects, with one person contributing domain knowledge and insights, while the other provides expertise in certain analysis methods and tools. Since then we have created training materials [39] and carried out training workshops to confirm that those materials and the software are easy for people to use independently. After the collaborative session with the epidemiologist, we discussed some of the insights with representatives from NHS Digital.

Patient and Public Involvement

Neither patients nor the public were involved in the design, or conduct, or reporting, or dissemination plans of this research.

RESULTS

The results are presented in a form that follows the workflow (Figure 1). First, the dataset was loaded and the set intersections were computed. There were 4371 intersections, a total of 960 million missing values, and all except 120 records were missing at least one value.

A value bar chart showed that, as expected, there were three monotone patterns of missing data – one for the diagnosis codes, one for the operation codes and one for the operation dates (Figure 2). However, the 4731 set intersections were many more than would occur if just those three monotone patterns were present, so we used combination count and length histograms to visualize the degree (number of missing fields) and cardinality (number of records) of the set intersections, respectively. This showed that 81% of the intersections involved 23 – 64 fields (Figure 4), and 83% of the intersections were rare (each involving < 0.1% of the records). Therefore, we did five iterations in the next stage of analysis (Figure 1 iteration (a)) to investigate the patterns of missing values in the general fields, diagnosis codes, operation codes, operation dates, and then operation codes and dates together. For each of those we performed one or more iterations to explain the patterns (Figure 1 iteration (b)).

General fields

For the general fields, a combination heatmap showed that the most common pattern was the 11,832,262 records that were just missing the SURVIVALTIME and AEKEY fields. The AEKEY links

For peer review only - http://bmjopen.bmj.com/site/about/guidelines.xhtml

APC data with hospital accident and emergency (A&E) data, so the pattern is consistent with the fact that most patients survive and do not enter hospital through A&E. However, on seeing the pattern the epidemiologist commented that the survival time should not be missing at all because the data provider had been asked to derive it for all patients based on the study census date, regardless of their mortality status. Clearly that had not been done, so she would have to estimate survival time aggregated to the nearest month in her survival analyses for those patients who did not die prior to the census date – thus losing precision of information compared with patients who had died prior to census. As well as loss of precision, this precluded analyses of short-term survival patterns within 30 days of admission.

From the value bar chart, the epidemiologist was also surprised to see EPIORDER and three date fields (MYADMIDATE, MYEPISTART and MYEPIEND) were sometimes missing instead of being complete (Figure 2). After selecting the two intersections that involved all three date fields from the heatmap, entropy calculations showed that all those records concerned maternity episodes (EPITYPE=5 "other delivery event" or EPITYPE=6 "birth event") and were the only records in the dataset with EPIORDER=98 ("not applicable"). EPIORDER was missing in three intersections (1210 records), and IGR calculations showed that the FAE and PROCODE fields were most likely to provide an explanation. Entropy calculations showed that EPIORDER was only missing for 10 health care providers and, for them, only when FAE=0 ("unfinished admission episode"). Unfinished episodes are common (3 million in the dataset), but EPIORDER was always provided by the other 553 health care providers.

Diagnosis codes

The diagnosis fields should have just exhibited a monotone pattern, with 20 intersections showing that the codes were missing progressively more often from DIAG_01 to DIAG_20. However, a combination heatmap showed a dramatically different picture, with a total of 158 intersections and all of the additional ones contained gaps in the diagnostic fields (Figure 3).

After interactively selecting the 138 unexpected intersections with gaps, another value bar chart showed that DIAG_03 was missing substantially more often than the subsequent diagnosis fields (Figure 5). Entropy calculations showed that all 1776 records in those intersections were for EPITYPE=3 ("birth episode"), all 1776 records were for ADMIMETH=31 ("admitted antepartum"), and 1575 of the records were from one PROCODE.

For peer review only - http://bmjopen.bmj.com/site/about/guidelines.xhtml

[Figure 5 should go approximately here]

Operation code and date fields

We first investigated the operation codes and dates separately, and then together. The code fields exhibited the same characteristics as the diagnosis codes. A combination heatmap showed that most (15,717,333) records exhibited a monotone pattern. However, there were also some gaps (72 set intersections; 2488 records). After we selected those intersections, IGR and entropy calculations showed that 1795 of the records belonged to ADMIMETH=31 and 1588 of the records were from the same PROCODE as the diagnosis gaps.

The operation date fields also exhibited the same characteristics. Most (15,716,187) records exhibited a monotone pattern, but gaps occurred in 89 intersections (3689 records). Again, entropy calculations showed that 2458 of the gaps occurred for ADMIMETH=31 and 2159 of the records were from same PROCODE as above.

Further unexpected patterns were revealed when the operation codes and dates were visualized together, because 45 intersections had no gaps but involved a different number of code and date fields. Each operation code should have a corresponding date in every record (i.e., a block missingness pattern), but 39 out of those 45 intersections contained fewer operation dates than codes, and the other six intersections contained extra dates.

The gaps in the diagnosis codes, and operation codes and dates fields were a surprise to the epidemiologist and to members of NHS Digital's Data Quality team, with whom we subsequently discussed the findings. Of particular concern to NHS Digital were the operation codes, because they affect the process that the NHS Digital Casemix Team use to calculate Healthcare Resource Groups (HRGs; the NHS equivalent of the diagnosis related groups that were pioneered in the USA). HRGs group together clinically similar treatments that involve similar levels of health care resource and, therefore, are one of the building blocks of the NHS's Payment by Results system for hospitals.[48] That system is used because it is much simpler than determining the amount to be paid for each of the tens of thousands of interventions and diagnoses that are used for patients in hospitals.

For peer review only - http://bmjopen.bmj.com/site/about/guidelines.xhtml

DISCUSSION

This study shows how set visualization techniques may be used to investigate and explain patterns of missing data in tabular data. Set visualization was applied by treating all of the records that were missing a given field as a set, so a set intersection comprised the records that were missing the same combination of fields. The two main visualization techniques were bar charts to show each set (i.e., the number of records missing each field) and heatmaps to show set intersections (i.e., the number of records that were only missing each specific combination fields but did have values for the other fields). They were combined with histograms and another bar chart to let the tool scale to the millions of records and thousands of set intersections in the data. The visualizations were essential for enabling users to see and reason about the completely unexpected patterns, and integrating the visualizations with two well-known data mining methods (IGR and entropy) enabled users to pinpoint some of the patterns' origins and explain the underlying structures. The interactive tool we developed is freely available.[39]

Bar charts, heatmaps and histograms are standard visualization techniques, of course. However, in the present study it was the way those techniques and the data mining methods were combined into an easy-to-use interactive tool that was novel, and key to being able to apply set visualization to find and explain the wide variety of patterns of missing data that occurred. To the authors' knowledge, the study is the first time that set visualization has been applied to large-scale EHR data and the study's strengths are: (a) showing the benefits of different set visualization techniques and how they combine to allow a holistic investigation of missing data patterns, and (b) the actionable insights that those visualizations produced for both researchers (the epidemiologist) and organisations (NHS Digital and individual hospitals).

In terms of missing data, most researchers and organisations only count the number of values that are missing in individual fields,[6 7 9 12 15-17] showing the results in either a table or the equivalent of our value bar chart. In our study that bar chart showed the high-level patterns that were dominant (the well-known monotone patterns in the diagnosis codes, operation codes and operation dates), but also and contrary to expectations that a few fields were missing some values instead of being complete.

The most important visualization was the combination heatmap, which we used to visualize set intersections. By using a heatmap, the unexpectedly large number of intersections and the multitude of gaps in the diagnosis codes, operation codes and operation dates all popped out (i.e., were immediately obvious) to users, and closer inspection revealed the discrepancies between pairs of operation codes

For peer review only - http://bmjopen.bmj.com/site/about/guidelines.xhtml

and dates. The tool's interactive data mining then enabled us to pinpoint the origin of some of the unexpected patterns. The few pieces of data quality software that can visualize set intersections are either interactive but only handle small datasets,[36] or generate static plots and necessitate users writing extra computer code to select specific set intersections (e.g., all those with gaps in the operation codes) for further investigation,[35 37 38] rather than interactively selecting those intersections with a few mouse clicks as with our ACE tool. Unlike ACE, none of that other software has any inbuilt data mining functionality.

The combination count and length histograms were useful during the first stage of analysis (Figure 1), to help a user gain a general understanding of the interwoven nature of the patterns of missing data. Had the combination heatmap been used at that stage then the user would have been overwhelmed by the 4371 rows. Our dataset only had 86 fields, but had there been more then the value count histogram would have been similarly useful during the first stage of analysis.

The study had two main limitations. First, the case study only involved one dataset, albeit one from a national organisation that provides similar datasets to many researchers and organisations each year. Second, the tool is primarily designed to work with single data tables. However, longitudinal data would be easy to accommodate by concatenating each dataset and adding a field that stored a date for each dataset. Missing data patterns that occurred throughout the data would be appear as patterns did in the present study, and patterns that were limited to a particular part(s) of the longitudinal data would be revealed by entropy calculations that involved the new field. Missing data patterns in a relational database could be investigated by either analysing each data table separately or joining fields of interest into a new table for analysis.

Our approach benefits analysts and researchers by making it easier to check data when it is received. In principle that would have allowed the epidemiologist to remedy the survival time issue by a data request amendment, but the time limit for doing that (one month) had passed before the case study started. Regarding the missing dates, as the tool pinpointed the origin as maternity records the epidemiologist made an informed decision to discard the records because she was conducting cardiovascular research. Our approach also benefits data providers by pinpointing where issues occur (e.g., the gaps originated almost entirely in a particular part of a specific hospital). Knowing that, it would be straightforward to provide feedback to rectify the problem via NHS Digital's existing data quality lifecycle,[12] Our approach helped communication within NHS Digital, allowing a connection to be made

For peer review only - http://bmjopen.bmj.com/site/about/guidelines.xhtml

between gaps in the operation code fields and the NHS's Payment by Results system [48] at a national level.

The present study showed how set visualization enables users to identify a variety of unexpected missing data patterns. With further research, that could be developed into a comprehensive library of patterns that users can apply in health data settings to analyse missing data faster and more comprehensively. Further research is also needed to determine the data mining methods that are best suited for determining the structures that explain each type of pattern, extending our use of IGR/entropy and the work of others with decision trees,[2]

CONCLUSIONS

Missing data has widespread impacts on secondary uses of EHRs. Investigations are typically limited to counting the number of values that are missing from key fields, and there is a lack of methods for investigating more complex patterns of missing values, and particularly patterns that are uncommon. A solution is offered by set visualization techniques, which treat records that are missing a given field as a set, and records that are missing multiple fields as set intersections. This study describes how interactive set visualization can be combined with data mining to identify and explain complex patterns of missing values. Using a case study with a 16-million record / 86 field EHR dataset, we demonstrate that set visualization revealed actionable insights that ranged from determining the data were not suitable for analysing short-term survival patterns, to providing feedback to hospitals to improve future data quality, and knock-on consequences for hospital payments.

Acknowledgements

The authors thank the NHS Digital staff who provided guidance during the research and comments about the implications of the findings.

Funding

This research was supported by the Engineering and Physical Sciences Research Council grant numbers EP/N013980/1 and EP/K503836/1, the British Heart Foundation grant number PG/13/81/30474, and the Alan Turing Institute.

For peer review only - http://bmjopen.bmj.com/site/about/guidelines.xhtml

Author contributions

MA and RAR designed the software. MA developed the software. MSH provided subject matter expertise about HES data. RAR wrote the first full version of the manuscript. All authors contributed to the case study and approved the final manuscript.

Competing interests

None.

Patient consent for publication

Not required.

Ethics approval

The study was approved by the University of Leeds MaPS and Engineering joint Faculty Research Ethics Committee (LIDA 15-002).

Data availability statement

The dataset was provided by NHS Digital (request number DARS-NIC-17649-G0X4B-v0.6) and, due to data governance restrictions, cannot be made openly available.

Provenance and peer review

Not commissioned; externally peer reviewed.

Figure captions

Figure 1 A workflow for investigating the patterns of missing values with ACE. The two levels of iteration are to: (a) select subsets of fields, (b) select groups of set intersections to explain a pattern in terms of the values of other fields.

Figure 2 A value bar chart showing the sets (the number of missing values in each field; NB: the Y-axis uses a log scale).

For peer review only - http://bmjopen.bmj.com/site/about/guidelines.xhtml

Figure 3 ACE utilises a tab pane layout with color-coded tabs (a). Each tab is split into a visualization panel (b) and a control panel (c), and the details depend on the type of visualization. This figure shows a heatmap of expected (d) and unexpected (e) set intersections that involve the DIAG_nn fields. A context menu (f) highlights various actions that can be performed on a selected intersection. The control panel contains a dataset summary (g), visualization size (h), heatmap legend (i), sorting methods (j), and filtering options (k).

Figure 4 A combination length histogram, which shows that most of the set intersections involved 23 – 64 fields.

Figure 5 A value bar chart showing the number of records in each set for the whole dataset (left) and the set intersections that, unexpectedly, contained gaps in the DIAG_nn fields (right).

REFERENCES

1. Khare R, Utidjian L, Ruth BJ, et al. A longitudinal analysis of data quality in a large pediatric data research network. *J Am Med Inform Assoc* 2017;24(6):1072-79.
2. Tierney NJ, Harden FA, Harden MJ, et al. Using decision trees to understand structure in missing data. *BMJ Open* 2015;5(6):e007450.
3. Collins GS, Reitsma JB, Altman DG, et al. Transparent reporting of a multivariable prediction model for individual prognosis or diagnosis (TRIPOD): the TRIPOD statement. *Journal of British Surgery* 2015;102(3):148-58.
4. Li R, Chen Y, Moore JH. Integration of genetic and clinical information to improve imputation of data missing from electronic health records. *J Am Med Inform Assoc* 2019;26(10):1056-63.
5. Kharrazi H, Wang C, Scharfstein D. Prospective EHR-based clinical trials: the challenge of missing data. *J Gen Intern Med* 2014;29:976-78.
6. Mathur R, Bhaskaran K, Chaturvedi N, et al. Completeness and usability of ethnicity data in UK-based primary care and hospital databases. *Journal of Public Health* 2014;36(4):684-92.
7. Murray J, Saxena S, Modi N, et al. Quality of routine hospital birth records and the feasibility of their use for creating birth cohorts. *Journal of Public Health* 2013;35(2):298-307.
8. Knight HE, Gurol-Urganci I, Mahmood TA, et al. Evaluating maternity care using national administrative health datasets: How are statistics affected by the quality of data on method of delivery? *BMC Health Services Research* 2013;13(1):200.
9. Smith M, Lix LM, Azimaee M, et al. Assessing the quality of administrative data for research: a framework from the Manitoba Centre for Health Policy. *J Am Med Inform Assoc* 2018;25(3):224-29.

For peer review only - http://bmjopen.bmj.com/site/about/guidelines.xhtml

10. The processing cycle and HES data quality: NHS Digital; 2022 [Available from: https://digital.nhs.uk/data-and-information/data-tools-and-services/data-services/hospital-episode-statistics/the-processing-cycle-and-hes-data-quality# accessed 13 May 2022.
11. Farquhar M. AHRQ quality indicators. Patient safety and quality: An evidence-based handbook for nurses: Agency for Healthcare Research and Quality (US) 2008.
12. HES data dictionary: Admitted patient care: NHS Digital; 2017 [Available from: http://content.digital.nhs.uk/media/25188/DD-APC-V10/pdf/DD-APC-V10.pdf accessed 13 May 2022.
13. Hospital Episode Statistics (HES): NHS Digital; 2019 [Available from: https://digital.nhs.uk/data-and-information/data-tools-and-services/data-services/hospital-episode-statistics accessed 13 May 2022.
14. Users, uses and access to Hospital Episode Statistics: NHS Digital; 2020 [Available from: https://digital.nhs.uk/data-and-information/data-tools-and-services/data-services/hospital-episode-statistics/users-uses-and-access-to-hospital-episode-statistics accessed 13 May 2022.
15. Goldstein BA, Navar AM, Pencina MJ, et al. Opportunities and challenges in developing risk prediction models with electronic health records data: a systematic review. *J Am Med Inform Assoc* 2017;24(1):198-208.
16. Herbert A, Wijlaars L, Zylbersztejn A, et al. Data resource profile: Hospital episode statistics admitted patient care (HES APC). *Int J Epidemiol* 2017;46(4):1093-93i.
17. Pringle M, Ward P, Chilvers C. Assessment of the completeness and accuracy of computer medical records in four practices committed to recording data on computer. *Br J Gen Pract* 1995;45(399):537-41.
18. Boslett AJ, Denham A, Hill EL, et al. Unclassified drug overdose deaths in the opioid crisis: emerging patterns of inequity. *J Am Med Inform Assoc* 2019;26(8-9):767-77.
19. Rubin DB. Characterizing the estimation of parameters in incomplete-data problems. *Journal of the American Statistical Association* 1974;69(346):467-74.
20. Enders KE. Applied missing data analysis. New York: Guilford 2010.
21. Monroe M, Lan R, Lee H, et al. Temporal event sequence simplification. *IEEE Trans Vis Comput Graphics* 2013;19:2227-36.
22. Caban JJ, Gotz D. Visual analytics in healthcare–opportunities and research challenges. *J Am Med Inform Assoc* 2015;22:260-62.
23. Wu DT, Chen AT, Manning JD, et al. Evaluating visual analytics for health informatics applications: a systematic review from the American Medical Informatics Association Visual Analytics Working Group Task Force on Evaluation. *J Am Med Inform Assoc* 2019;26(4):314-23.
24. Rind A, Wang TD, Wolfgang A, et al. Interactive information visualization to explore and query electronic health records. *Foundations and Trends in Human-Computer Interaction* 2013;5:207-98.
25. West VL, Borland D, Hammond WE. Innovative information visualization of electronic health record data: a systematic review. *J Am Med Inform Assoc* 2015;22:330-39.
26. Dowding D, Randell R, Gardner P, et al. Dashboards for improving patient care: Review of the literature. *Int J Med Inf* 2015;84(2):87-100.
27. Alper B, Riche N, Ramos G, et al. Design study of LineSets, a novel set visualization technique. *IEEE Trans Vis Comput Graphics* 2011;17(12):2259-67.
28. Dörk M, Riche NH, Ramos G, et al. Pivotpaths: Strolling through faceted information spaces. *IEEE Trans Vis Comput Graphics* 2012;18(12):2709-18.
29. Freiler W, Matkovic K, Hauser H. Interactive visual analysis of set-typed data. *IEEE Trans Vis Comput Graphics* 2008;14(6):1340-47.
30. Noselli M, Mason D, Mohammed MA, et al. MonAT: A Visual Web-based Tool to Profile Health Data Quality. HEALTHINF2017:26-34.

For peer review only - http://bmjopen.bmj.com/site/about/guidelines.xhtml

31. Yalcin MA, Elmqvist N, Bederson BB. AggreSet: Rich and scalable set exploration using visualizations of element aggregations. *IEEE Trans Vis Comput Graphics* 2015;22(1):688-97.
32. OpenRefine: A free, open source, powerful tool for working with messy data [program], 2018. Available from: http://openrefine.org/ accessed 13 May 2022.
33. Kandel S, Parikh R, Paepcke A, et al. Profiler: integrated statistical analysis and visualization for data quality assessment. Proceedings of the International Working Conference on Advanced Visual Interfaces: ACM 2012:547-54.
34. Trifacta Wrangler [program], 2020. Available from: https://www.trifacta.com/ accessed 13 May 2022.
35. Templ M, Alfons A, Filzmoser P. Exploring incomplete data using visualization techniques. *Advances in Data Analysis and Classification* 2012;6(1):29-47.
36. Lex A, Gehlenborg N, Strobelt H, et al. UpSet: visualization of intersecting sets. *IEEE Trans Vis Comput Graphics* 2014;20(12):1983-92.
37. Conway JR, Lex A, Gehlenborg N. UpSetR: an R package for the visualization of intersecting sets and their properties. *Bioinformatics* 2017;33(18):2938-40.
38. UpSetPlot [program], 2021. Available from: https://pypi.org/project/UpSetPlot/ accessed 13 May 2022.
39. The ACE software, and training materials for visualizing missing data and set-type data [program], 2022. Available from: https://doi.org/10.5518/1133 accessed 13 May 2022.
40. HyperSQL Homepage [program]. 2.5.0 version, 2019. Available from: http://hsqldb.org/ accessed 13 May 2022.
41. Arbesser C, Spechtenhauser F, Mühlbacher T, et al. Visplause: Visual data quality assessment of many time series using plausibility checks. *IEEE Trans Vis Comput Graphics* 2016;23(1):641-50.
42. Tierney NJ, Cook DH. Expanding tidy data principles to facilitate missing data exploration, visualization and assessment of imputations. *arXiv preprint arXiv:180902264* 2018
43. Menon S, Zaidi E. Market guide for data preparation tools 2019 [Available from: https://www.gartner.com/en/documents/3906957/market-guide-for-data-preparation-tools accessed 13 May 2022.
44. Honaker J, King G, Blackwell M. Amelia II: A program for missing data. *Journal of Statistical Software* 2011;45:1-47.
45. Matrix reordering methods for table and network visualization. Computer Graphics Forum; 2016. Wiley Online Library.
46. Quinlan JR. Induction of decision trees. *Machine Learning* 1986;1(1):81-106.
47. Shannon CE. A mathematical theory of communication. *ACM SIGMOBILE Mobile Computing and Communications Review* 2001;5(1):3-55.
48. A simple guide to payment by results: Department of Health; 2012 [Available from: https://assets.publishing.service.gov.uk/government/uploads/system/uploads/attachment_data/file/213150/PbR-Simple-Guide-FINAL.pdf accessed 13 May 2022.

For peer review only - http://bmjopen.bmj.com/site/about/guidelines.xhtml

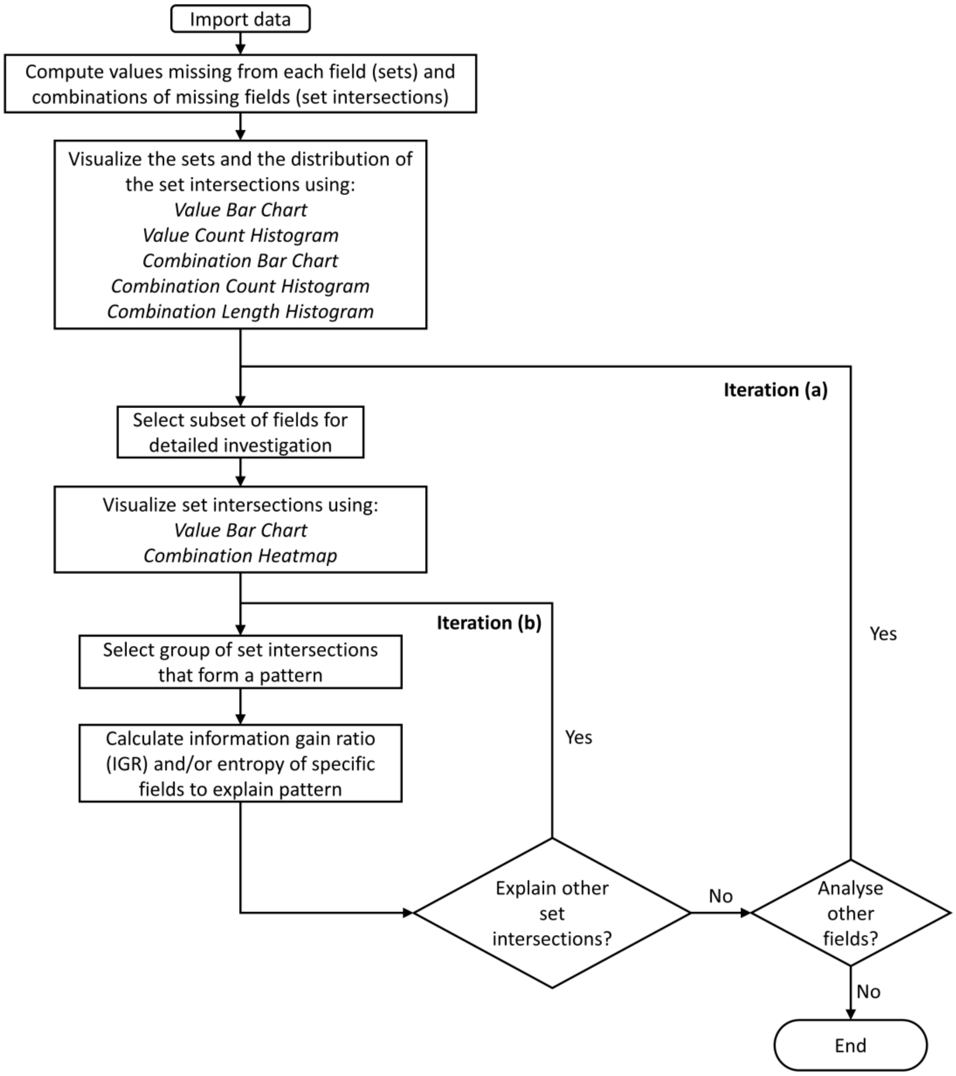

Figure 1 A workflow for investigating the patterns of missing values with ACE. The two levels of iteration are to: (a) select subsets of fields, (b) select groups of set intersections to explain a pattern in terms of the values of other fields.

1000x1119mm (96 x 96 DPI)

For peer review only - http://bmjopen.bmj.com/site/about/guidelines.xhtml

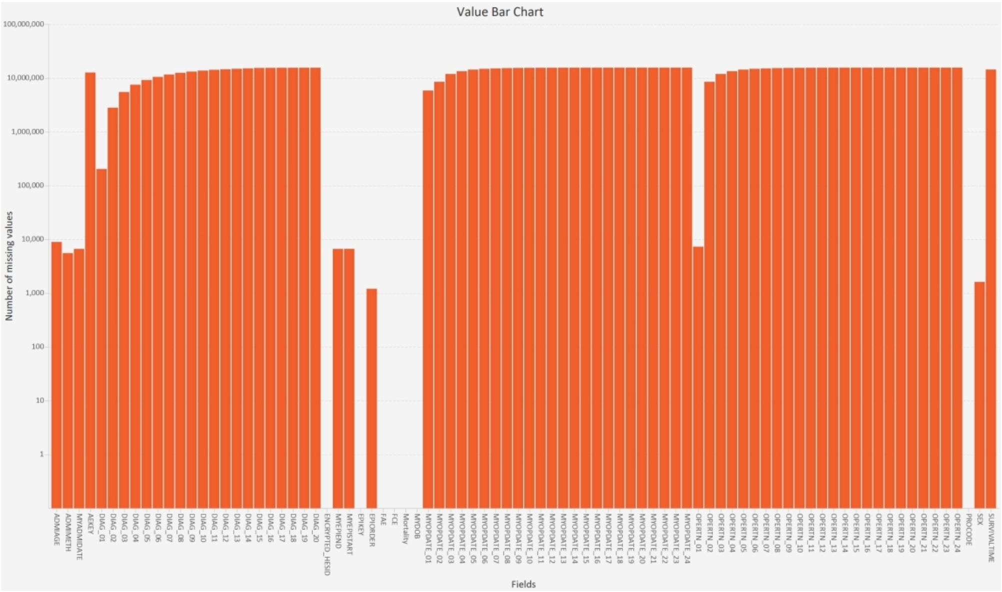

Figure 2 A value bar chart showing the sets (the number of missing values in each field; NB: the Y-axis uses a log scale).

567x333mm (144 x 144 DPI)

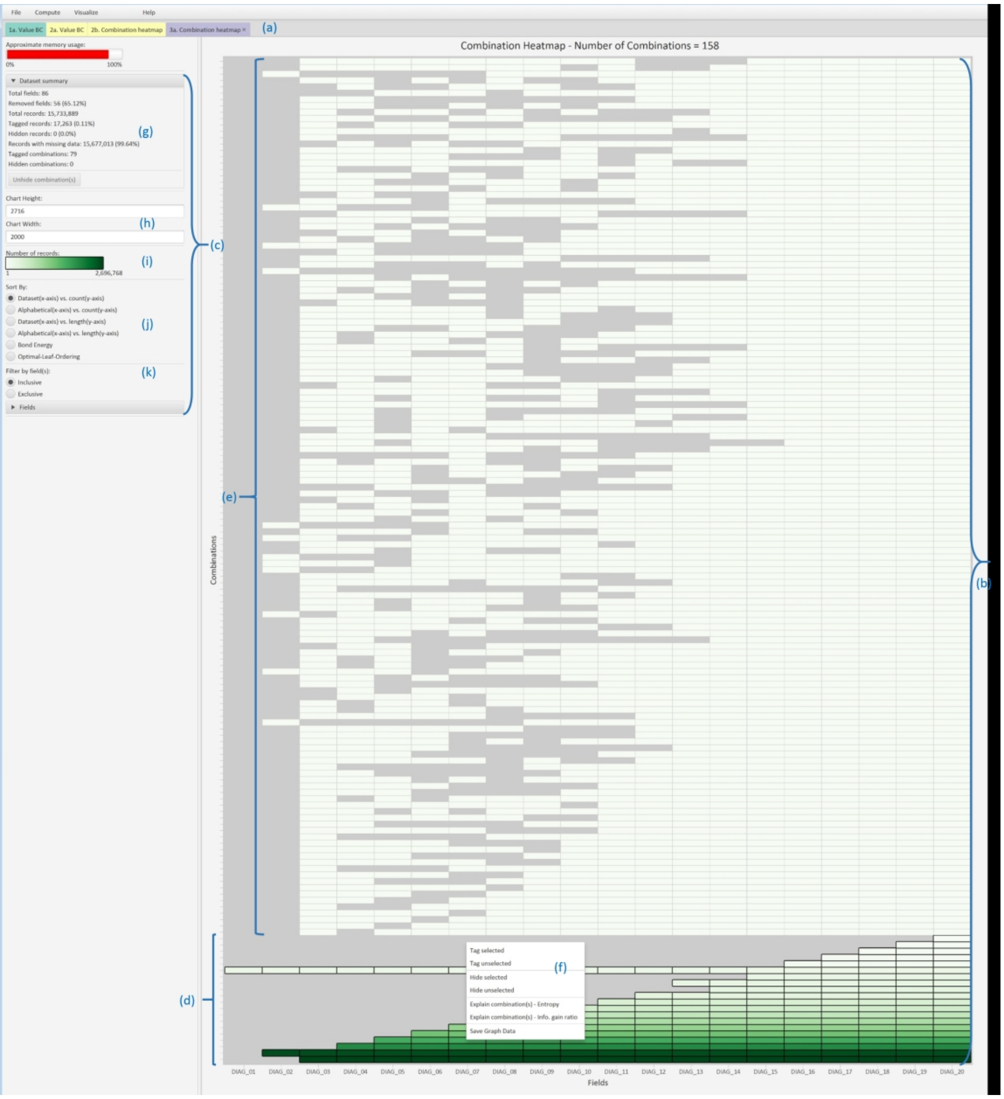

Figure 3 ACE utilises a tab pane layout with color-coded tabs (a). Each tab is split into a visualization panel (b) and a control panel (c), and the details depend on the type of visualization. This figure shows a heatmap of expected (d) and unexpected (e) set intersections that involve the DIAG_nn fields. A context menu (f) highlights various actions that can be performed on a selected intersection. The control panel contains a dataset summary (g), visualization size (h), heatmap legend (i), sorting methods (j), and filtering options (k).

1278x1392mm (59 x 59 DPI)

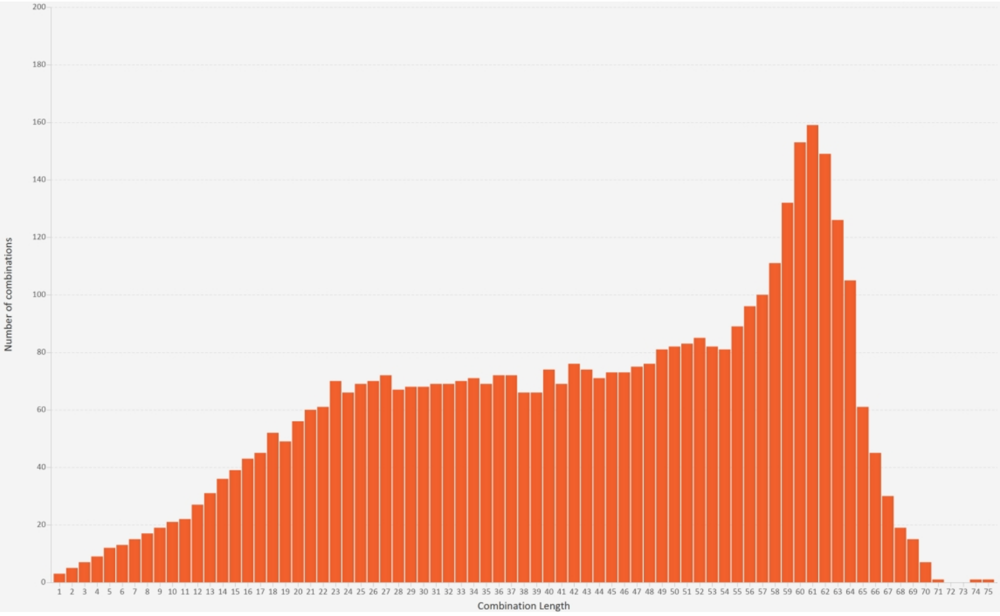

Figure 4 A combination length histogram, which shows that most of the set intersections involved 23 – 64 fields.

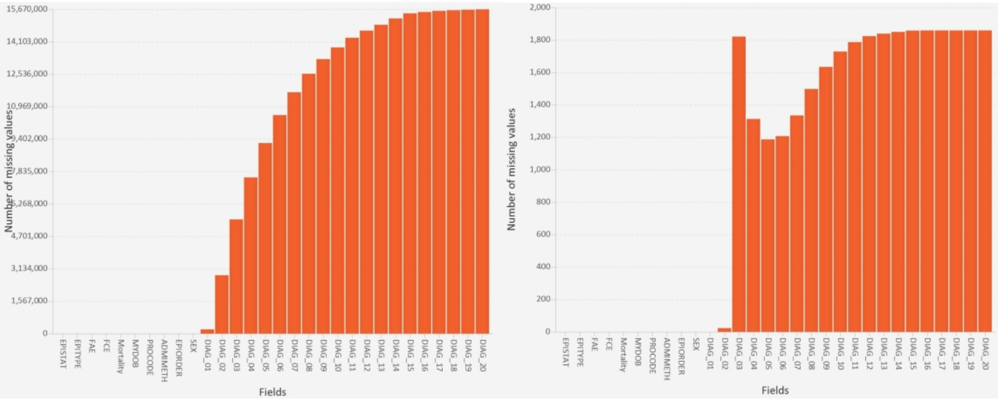

Figure 5 A value bar chart showing the number of records in each set for the whole dataset (left) and the set intersections that, unexpectedly, contained gaps in the DIAG_nn fields (right).

507x200mm (144 x 144 DPI)

Reporting checklist for prediction model development/validation.

Based on the TRIPOD guidelines.

Instructions to authors

Complete this checklist by entering the page numbers from your manuscript where readers will find each of the items listed below.

Your article may not currently address all the items on the checklist. Please modify your text to include the missing information. If you are certain that an item does not apply, please write "n/a" and provide a short explanation.

Upload your completed checklist as an extra file when you submit to a journal.

In your methods section, say that you used the TRIPODreporting guidelines, and cite them as:

Collins GS, Reitsma JB, Altman DG, Moons KG. Transparent reporting of a multivariable prediction model for individual prognosis or diagnosis (TRIPOD): The TRIPOD statement.

A note to the reviewers: We have used the TRIPOD reporting guidelines, because that is better-suited to this type of research than other guidelines. However, it should be noted that TRIPOD is designed for prediction model studies rather than those that develop visualization software, which is why we have marked a number of the guidelines as n/a (particularly those that are specifically about prediction itself or the statistical analysis methods). For some guidelines we have added comments as well as the page number, to help the reader.

Reporting Item	Page Number

For peer review only

**Title**			
	#1	Identify the study as developing and / or validating a multivariable prediction model, the target population, and the outcome to be predicted.	1 (NB: the study developed a visualization tool, rather than a statistical model)
**Abstract**			
	#2	Provide a summary of objectives, study design, setting, participants, sample size, predictors, outcome, statistical analysis, results, and conclusions.	3
**Introduction**			
	#3a	Explain the medical context (including whether diagnostic or prognostic) and rationale for developing or validating the multivariable prediction model, including references to existing models.	4 (and p5/6 for references to existing tools)
	#3b	Specify the objectives, including whether the study describes the development or validation of the model or both.	4
**Methods**			
Source of data	#4a	Describe the study design or source of data (e.g., randomized trial, cohort, or registry data), separately for the	6

For peer review only

		development and validation data sets, if applicable.	
Source of data	#4b	Specify the key study dates, including start of accrual; end of accrual; and, if applicable, end of follow-up.	n/a
Participants	#5a	Specify key elements of the study setting (e.g., primary care, secondary care, general population) including number and location of centres.	6
Participants	#5b	Describe eligibility criteria for participants.	6
Participants	#5c	Give details of treatments received, if relevant	n/a
Outcome	#6a	Clearly define the outcome that is predicted by the prediction model, including how and when assessed.	n/a
Outcome	#6b	Report any actions to blind assessment of the outcome to be predicted.	n/a
Predictors	#7a	Clearly define all predictors used in developing or validating the multivariable prediction model, including how and when they were measured	n/a

For peer review only

Predictors	#7b	Report any actions to blind assessment of predictors for the outcome and other predictors.	n/a
Sample size	#8	Explain how the study size was arrived at.	6 (to demonstrate scalability to large datasets, the study used a whole year of NHS APC data, apart from the exclusion criteria described on p6)
Missing data	#9	Describe how missing data were handled (e.g., complete-case analysis, single imputation, multiple imputation) with details of any imputation method.	7-8 (described in the tool's design)
Statistical analysis methods	#10a	If you are developing a prediction model describe how predictors were handled in the analyses.	n/a
Statistical analysis methods	#10b	If you are developing a prediction model, specify type of model, all model-building procedures (including any predictor selection), and method for internal validation.	n/a
Statistical analysis methods	#10c	If you are validating a prediction model, describe how the predictions were calculated.	n/a

For peer review only

Statistical analysis methods	#10d	Specify all measures used to assess model performance and, if relevant, to compare multiple models.	n/a
Statistical analysis methods	#10e	If you are validating a prediction model, describe any model updating (e.g., recalibration) arising from the validation, if done	n/a
Risk groups	#11	Provide details on how risk groups were created, if done.	n/a
Development vs. validation	#12	For validation, identify any differences from the development data in setting, eligibility criteria, outcome, and predictors.	n/a
**Results**			
Participants	#13a	Describe the flow of participants through the study, including the number of participants with and without the outcome and, if applicable, a summary of the follow-up time. A diagram may be helpful.	9 (the analysis workflow)
Participants	#13b	Describe the characteristics of the participants (basic demographics, clinical features, available predictors), including	9 (the quantity of missing values)

For peer review only

		the number of participants with missing data for predictors and outcome.	
Participants	#13c	For validation, show a comparison with the development data of the distribution of important variables (demographics, predictors and outcome).	n/a
Model development	#14a	If developing a model, specify the number of participants and outcome events in each analysis.	9-11 (the Results is divided into three sections, which each describe the outcome for that part of the analysis)
Model development	#14b	If developing a model, report the unadjusted association, if calculated between each candidate predictor and outcome.	n/a
Model specification	#15a	If developing a model, present the full prediction model to allow predictions for individuals (i.e., all regression coefficients, and model intercept or baseline survival at a given time point).	n/a
Model specification	#15b	If developing a prediction model, explain how to the use it.	n/a
Model performance	#16	Report performance measures (with CIs) for the prediction model.	n/a

For peer review only

Model-updating	#17	If validating a model, report the results from any model updating, if done (i.e., model specification, model performance).	n/a
**Discussion**			
Limitations	#18	Discuss any limitations of the study (such as nonrepresentative sample, few events per predictor, missing data).	13
Interpretation	#19a	For validation, discuss the results with reference to performance in the development data, and any other validation data	n/a
Interpretation	#19b	Give an overall interpretation of the results, considering objectives, limitations, results from similar studies, and other relevant evidence.	12
Implications	#20	Discuss the potential clinical use of the model and implications for future research	13
**Other information**			
Supplementary information	#21	Provide information about the availability of supplementary resources, such as	12 (the tool is freely available; DOI in the references)

For peer review only

		study protocol, Web calculator, and data sets.	
Funding	#22	Give the source of funding and the role of the funders for the present study.	14

None The TRIPOD checklist is distributed under the terms of the Creative Commons Attribution License CC-BY. This checklist can be completed online using https://www.goodreports.org/, a tool made by the EQUATOR Network in collaboration with Penelope.ai

For peer review only

BMJ Open

Using set visualization to find and explain patterns of missing values: A case study with NHS hospital episode statistics data

Journal:	*BMJ Open*
Manuscript ID	bmjopen-2022-064887.R1
Article Type:	Original research
Date Submitted by the Author:	17-Oct-2022
Complete List of Authors:	Ruddle, Roy; University of Leeds, School of Computing and Leeds Institute for Data Analytics<br>Adnan, Muhammad; Higher Colleges of Technology, Computer Science<br>Hall, Marlous ; University of Leeds, Institute of Cardiovascular & Metabolic Medicine and Leeds Institute for Data Analytics
**Primary Subject Heading**:	Health informatics
Secondary Subject Heading:	Qualitative research
Keywords:	Health informatics < BIOTECHNOLOGY & BIOINFORMATICS, STATISTICS & RESEARCH METHODS, QUALITATIVE RESEARCH

SCHOLARONE™
Manuscripts

I, the Submitting Author has the right to grant and does grant on behalf of all authors of the Work (as defined in the below author licence), an exclusive licence and/or a non-exclusive licence for contributions from authors who are: i) UK Crown employees; ii) where BMJ has agreed a CC-BY licence shall apply, and/or iii) in accordance with the terms applicable for US Federal Government officers or employees acting as part of their official duties; on a worldwide, perpetual, irrevocable, royalty-free basis to BMJ Publishing Group Ltd ("BMJ") its licensees and where the relevant Journal is co-owned by BMJ to the co-owners of the Journal, to publish the Work in this journal and any other BMJ products and to exploit all rights, as set out in our licence.

The Submitting Author accepts and understands that any supply made under these terms is made by BMJ to the Submitting Author unless you are acting as an employee on behalf of your employer or a postgraduate student of an affiliated institution which is paying any applicable article publishing charge ("APC") for Open Access articles. Where the Submitting Author wishes to make the Work available on an Open Access basis (and intends to pay the relevant APC), the terms of reuse of such Open Access shall be governed by a Creative Commons licence – details of these licences and which Creative Commons licence will apply to this Work are set out in our licence referred to above.

Other than as permitted in any relevant BMJ Author's Self Archiving Policies, I confirm this Work has not been accepted for publication elsewhere, is not being considered for publication elsewhere and does not duplicate material already published. I confirm all authors consent to publication of this Work and authorise the granting of this licence.

For peer review only

Title: Using set visualization to find and explain patterns of missing values: A case study with NHS hospital episode statistics data

Authors

Corresponding author: Roy A. Ruddle, School of Computing and Leeds Institute for Data Analytics, University of Leeds, Leeds, LS2 9NL, UK, r.a.ruddle@leeds.ac.uk

Muhammad Adnan, Higher Colleges of Technology, University City, Sharjah, UAE, madnan1@hct.ac.ae

Marlous Hall, Leeds Institute of Cardiovascular & Metabolic Medicine and Leeds Institute for Data Analytics, University of Leeds, Leeds, UK, m.s.hall@leeds.ac.uk

For peer review only

For peer review only - http://bmjopen.bmj.com/site/about/guidelines.xhtml

I, the Submitting Author has the right to grant and does grant on behalf of all authors of the Work (as defined in the below author licence), an exclusive licence and/or a non-exclusive licence for contributions from authors who are: i) UK Crown employees; ii) where BMJ has agreed a CC-BY licence shall apply, and/or iii) in accordance with the terms applicable for US Federal Government officers or employees acting as part of their official duties; on a worldwide, perpetual, irrevocable, royalty-free basis to BMJ Publishing Group Ltd ("BMJ") its licensees and where the relevant Journal is co-owned by BMJ to the co-owners of the Journal, to publish the Work in BMJ Open and any other BMJ products and to exploit all rights, as set out in our licence.

The Submitting Author accepts and understands that any supply made under these terms is made by BMJ to the Submitting Author unless you are acting as an employee on behalf of your employer or a postgraduate student of an affiliated institution which is paying any applicable article publishing charge ("APC") for Open Access articles. Where the Submitting Author wishes to make the Work available on an Open Access basis (and intends to pay the relevant APC), the terms of reuse of such Open Access shall be governed by a Creative Commons licence – details of these licences and which Creative Commons licence will apply to this Work are set out in our licence referred to above.

For peer review only

For peer review only - http://bmjopen.bmj.com/site/about/guidelines.xhtml

ABSTRACT

Objectives: Missing data is the most common data quality issue in electronic health records (EHRs). Missing data checks implemented in common analytical software are typically limited to counting the number of missing values in individual fields, but researchers and organisations also need to understand multi-field missing data patterns to better inform advanced missing data strategies for which counts or numerical summaries are poorly suited. This study shows how set-based visualization enables multi-field missing data patterns to be discovered and investigated.

Design: Development and evaluation of interactive set visualization techniques to find patterns of missing data and generate actionable insights. The visualizations comprised easily interpretable bar charts for sets, heatmaps for set intersections and histograms for distributions of both sets and intersections.

Setting and participants: Anonymised Admitted Patient Care health records for NHS hospitals and independent sector providers in England. The visualization and data mining software was run over 16 million records and 86 fields in the dataset.

Results: The dataset contained 960 million missing values. Set visualization bar charts showed how those values were distributed across the fields, including several fields that, unexpectedly, were not complete. Set intersection heatmaps revealed unexpected gaps in diagnosis, operation and date fields because diagnosis and operation fields were not filled up sequentially and some operations did not have corresponding dates. Information gain ratio and entropy calculations allowed us to identify the origin of each unexpected pattern, in terms of the values of other fields.

Conclusions: Our findings show how set visualization reveals important insights about multi-field missing data patterns in large EHR datasets. The study revealed both rare and widespread data quality issues that were previously unknown, and allowed a particular part of a specific hospital to be pinpointed as the origin of rare issues that NHS Digital did not know exist.

Keywords: Data visualization; data quality; missing values; electronic health records

Word count: 4,237

For peer review only - http://bmjopen.bmj.com/site/about/guidelines.xhtml

ARTICLE SUMMARY

Strengths and limitations of this study

- This study demonstrates the utility of interactive set visualization techniques for finding and explaining patterns of missing values, irrespective of whether those patterns are common or rare.
- Techniques were evaluated with a large national admitted patient care dataset (16-million records across 86 fields)
- Evaluation only involved one single-table dataset, but that was from a national organisation providing many similar data extracts each year to researchers and organisations.
- Evaluation of the interactive set visualization techniques did not involve formal usability testing.

INTRODUCTION

Missing data occurs more often in electronic health records (EHRs) than any other single data quality issue.[1] The motivating problem for the present research was how to investigate patterns of missing data that involve many variables and explain those patterns by identifying the underlying structures.[2] Our objective was to develop and evaluate a method to achieve that using set visualization, to enable users to generate actionable insights from datasets that contain millions of records. To be successful, the method needs to reveal both known and unknown patterns, including those that are often obscured in a dataset due to their rarity. To help ensure that the visualization method was scalable we chose a set-based approach, but limited the scope of the research to analysing flat file data tables. The research used the Transparent reporting of a multivariable prediction model for individual prognosis or diagnosis (TRIPOD) reporting guidelines.[3]

Researchers need to investigate patterns of missing values to understand possible effects on cohort selection, bias, impact on data linkages and to design appropriate missing data techniques such as multiple imputation by chained equations.[1 4-8] Organisations collecting and providing such data strive to continuously improve data quality.[9-12] We collaborate with one such organisation, NHS Digital, which is the UKs national information and technology service collecting more than 100 million records/year of Hospital Episode Statistics (HES) [13] for the NHS in England. HES data are directly used in the payment of hospitals throughout England, as well as for secondary uses such as research, quality indicators and planning health services.[8 14]

For peer review only - http://bmjopen.bmj.com/site/about/guidelines.xhtml

At present, the only checks that organisations typically perform are basic counts of missing values in individual fields,[6 7 9 12 15-17] with researchers then implementing a range of techniques from removing records with missing values, dropping variables or more advanced analytical techniques including multiple imputation by chained equations and bootstrapping integrated into their analyses.[4 7 15] In NHS Digital's case those checks are limited to core data fields (e.g., NHS Number), and if the percentage missing exceeds a threshold (30%) then feedback is sent to the hospital.[10 12] However, researchers also want to investigate patterns of missing values that involve multiple fields,[1 18] and NHS Digital wants to do that to define new business rules for data cleaning and to cross-reference variations in data quality with external influences (e.g. changes in policy or coding improvement initiatives).

Patterns of missing values may be computed and/or visualized.[2] Some computations model the distribution of missing values to estimate whether they adhere to a predefined pattern and to allow the analysts to consider assumptions about missing at random (MAR), missing completely at random (MCAR) or missing not at random patterns (MNAR [19]), with the latter including monotone and unit non-response ("block") patterns.[20] However these methods rely on probabilistic models, so by their very nature, they are poorly suited for detecting rare patterns of missingness.

An alternative approach is to use set analysis. Imagine a dataset has three fields (A, B and C). Set A is all of the records that are missing the value for Field A, and similarly for sets B and C. There are four possible exclusive set intersections (AB, AC, BC and ABC), where intersection AB contains the records that are only missing values for Fields A and B, and so on. The number of intersections cannot be greater than the number of records in the dataset,[21] so it is computationally tractable to identify every combination of fields that are missing together irrespective of whether it occurs millions of time or only once.

Sophisticated visualization tools and techniques have been developed for cohort selection, risk analysis and other types of detailed EHR data analysis,[22-24] but data quality use cases are a notable omission [25]. Visualization dashboards are widely used in healthcare,[26] but focus on high-level metrics such as the number of values that are missing from key fields, which corresponds to visualizing specific sets of missing data. However, it is not sufficient for tools to visualize sets of missing data because users need to visualize set intersections if they are to investigate multi-field patterns of missing values in detail. Most tools to date adopt designs that are unsuitable for showing intersections that involve many sets

For peer review only - http://bmjopen.bmj.com/site/about/guidelines.xhtml

because Venn diagram or node-link representations are used, [27-29] only display pairwise intersections,[30] [31] or require excessive interaction because users have to select specific fields of interest.[32-34]

The most appropriate technique for visualizing set intersections is a matrix of sets vs. intersections. That technique is provided by VIM,[35] but only as static plots. UpSet [36] provides interactive set intersection visualization but, when the present research was conducted, the latest version (UpSet2) only allowed 5 MB of data to be loaded and our dataset was one thousand times larger. Since then, more scalable R and Python UpSet implementations have been released [37] [38] but, like VIM they also generate static plots.

MATERIALS AND METHODS

Setting and dataset

We used a 15,733,889 record extract of Admitted Patient Care (APC) data from 2016-17 hospital episode statistics (HES) from NHS Digital. APC data contains information about admissions to NHS hospitals in England and to independent sector providers paid for by the NHS, and is collected for clinical uses, determining payments to hospitals, care quality reports and a wide variety of research [16].

Our extract was from a project that was studying long-term outcomes and hospitalisation rates for acute myocardial infarction (heart attack) patients, and requested help investigating the quality of their data. The cohort comprised patients who were aged 18 or older at 31st March 2009, and had not had an acute myocardial infarction during years 2001/02 to 2007/08.

The dataset was processed to flag any "unknown" values in each field [12] as missing, and had 86 fields that may be divided into four groups. Twenty fields contained ICD-10 diagnosis codes for a patient's illness or condition (DIAG_01 to DIAG_20), which should be filled in incrementally and exhibit a monotone pattern of missing data. Similarly, 24 fields contained codes about a patient's operations (OPERTN_01 to OPERTN_24) and 24 fields contained the corresponding dates for those operations (MYOPDATE_01 to MYOPDATE_24), with the operations and dates fields exhibiting monotone patterns, and each pair of operation/date fields exhibiting a block missingness pattern. There were 18 general fields: patient, episode and A&E record identifiers (ENCRYPTED_HESID; EPIKEY; AEKEY), episode order, status and type (EPIORDER; EPISTAT; EPITYPE), flags to indicate a finished admission and consultant episode (FAE; FCE), dates of admission, episode start and episode end (MYADMIDATE; MYEPISTART; MYEPIEND), patient date of birth, age and sex (MYDOB; ADMIAGE;

For peer review only - http://bmjopen.bmj.com/site/about/guidelines.xhtml

SEX), whether patient was alive or dead and time from episode end date to death (Mortality; SURVIVALTIME), admission method (ADMIMETH) and the hospital's code (PROCODE).

Visualization design

We designed a novel tool called ACE (Analysis of Combinations of Events), which is freely available for download and use.[39] The tool reads data from a flat file format (a CSV or other text file), calculates the exclusive set intersections, and stores the results in a memory-resident HyperSQL database.[40] ACE is implemented in Java and R, with a JavaFX graphical user interface.

ACE combines set-based visualization and data mining methods in an integrated, interactive tool that enables users to investigate multi-field missing data patterns. Figure 1 shows a recommended workflow, which starts with computing the sets (one for each field) and set intersections (each combination of missing fields), and then visualizing the sets (the number of values missing from each field) and distributions of the set intersections (to investigate high-level patterns). The substance of the workflow follows, with two levels of iteration.

[Figure 1 should go approximately here]

The user interface is based on a tab pane layout that allows users to: (1) see each iterative step taken during the analysis, (2) undo steps by closing tabs, and (3) compare patterns of missing values in different subsets of the data by switching tabs. An alternative to tabs would have been to integrate all of the visualizations into a single window, but that would have reduced the screen space available for each visualization and limit the detail that users could see without scrolling or zooming.

Two of the visualizations are for sets and four visualizations are for set intersections. The primary set visualization is called a *value bar chart* (Figure 2), which shows the number of missing values in each field (this is standard functionality in other tools [30-37 41-43]). That visualization is complemented by a *value count histogram*, which calculates bins for the number of missing values, shows the number of fields in each bin, and lets our tool scale to datasets with an unlimited number of fields. Both of the visualizations allow users to sort the fields, and to select certain fields for the next step in the analysis.

[Figure 2 should go approximately here]

For peer review only - http://bmjopen.bmj.com/site/about/guidelines.xhtml

The primary set intersection visualization is called a *combination heatmap* (Figure 3), which shows fields (X axis) × set intersections (Y axis) and the number of records that are in each intersection (colour). The height of the heatmap depends on the number of intersections, which makes it suitable for large datasets. By contrast, a missingness map [44] displays a separate row for each record in a dataset, so the case study's 16 million record dataset would produce a map with 16 million rows, which is clearly not practical.

[Figure 3 should go approximately here]

The heatmap fields may be sorted alphabetically or into dataset order, the intersections may be sorted by degree (number of missing fields) and cardinality (number of records), and the heatmap cells may be clustered using two heuristic sorting methods from a recent state-of-the-art report.[45] The heatmap is complemented by a *combination count histogram* and a *combination length histogram* (Figure 4) that calculate bins for the number of records and fields, respectively, and show the number of intersections in each bin. This lets ACE scale to an unlimited number of set intersections. The sixth visualization (a *combination bar chart*) is for intermediate scalability because it shows the number of records in each intersection.

[Figure 4 should go approximately here]

The tool's data mining methods comprise information gain ratio (IGR) [46] and entropy calculations, which are key concepts in information theory,[47] IGR measures the change in entropy when a field is used to classify data, which enables users to rank fields in terms of their ability at explaining certain intersections. Entropy quantifies how cleanly data is divided into different classes, which enables users to identify which values of a specific field are strongly associated with the intersections. IGR output was displayed in a bar chart in its own tab, and entropy output was displayed in a bar chart or table in its own tab.

All of the above visualizations and data mining techniques can be applied to either the whole dataset or subsets of it, chosen by selecting or filtering-out fields and/or combinations. This lets users iteratively analyse multiple patterns of missing values that coexist in the dataset.

For peer review only - http://bmjopen.bmj.com/site/about/guidelines.xhtml

Case study

The ACE software was evaluated in a case study with a senior epidemiologist who was studying long-term outcomes and hospitalisation rates for survivors of acute myocardial infarction. During the case study, the epidemiologist and one of the software's developers worked together, with the latter controlling the user interface. This mimics the collaborative working environment that is found in many data science projects, with one person contributing domain knowledge and insights, while the other provides expertise in certain analysis methods and tools. Since then we have created training materials [39] and carried out training workshops to confirm that those materials and the software are easy for people to use independently. After the collaborative session with the epidemiologist, we discussed some of the insights with representatives from NHS Digital.

Patient and Public Involvement

Neither patients nor the public were involved in the design, or conduct, or reporting, or dissemination plans of this research.

RESULTS

The results are presented in a form that follows the workflow (Figure 1). First, the dataset was loaded and the set intersections were computed. There were 4371 intersections, a total of 960 million missing values, and all except 120 records were missing at least one value.

A value bar chart showed that, as expected, there were three monotone patterns of missing data – one for the diagnosis codes, one for the operation codes and one for the operation dates (Figure 2). However, the 4731 set intersections were many more than would occur if just those three monotone patterns were present, so we used combination count and length histograms to visualize the degree (number of missing fields) and cardinality (number of records) of the set intersections, respectively. This showed that 81% of the intersections involved 23 – 64 fields (Figure 4), and 83% of the intersections were rare (each involving < 0.1% of the records). Therefore, we did five iterations in the next stage of analysis (Figure 1 iteration (a)) to investigate the patterns of missing values in the general fields, diagnosis codes, operation codes, operation dates, and then operation codes and dates together. For each of those we performed one or more iterations to explain the patterns (Figure 1 iteration (b)).

General fields

For the general fields, a combination heatmap showed that the most common pattern was the 11,832,262 records that were just missing the SURVIVALTIME and AEKEY fields. The AEKEY links

For peer review only - http://bmjopen.bmj.com/site/about/guidelines.xhtml

APC data with hospital accident and emergency (A&E) data, so the pattern is consistent with the fact that most patients survive and do not enter hospital through A&E. However, on seeing the pattern the epidemiologist commented that the survival time should not be missing at all because the data provider had been asked to derive it for all patients based on the study census date, regardless of their mortality status. Clearly that had not been done, so she would have to estimate survival time aggregated to the nearest month in her survival analyses for those patients who did not die prior to the census date – thus losing precision of information compared with patients who had died prior to census. As well as loss of precision, this precluded analyses of short-term survival patterns within 30 days of admission.

From the value bar chart, the epidemiologist was also surprised to see EPIORDER and three date fields (MYADMIDATE, MYEPISTART and MYEPIEND) were sometimes missing instead of being complete (Figure 2). After selecting the two intersections that involved all three date fields from the heatmap, entropy calculations showed that all those records concerned maternity episodes (EPITYPE=5 "other delivery event" or EPITYPE=6 "birth event") and were the only records in the dataset with EPIORDER=98 ("not applicable"). EPIORDER was missing in three intersections (1210 records), and IGR calculations showed that the FAE and PROCODE fields were most likely to provide an explanation. Entropy calculations showed that EPIORDER was only missing for 10 health care providers and, for them, only when FAE=0 ("unfinished admission episode"). Unfinished episodes are common (3 million in the dataset), but EPIORDER was always provided by the other 553 health care providers.

Diagnosis codes

The diagnosis fields should have just exhibited a monotone pattern, with 20 intersections showing that the codes were missing progressively more often from DIAG_01 to DIAG_20. However, a combination heatmap showed a dramatically different picture, with a total of 158 intersections and all of the additional ones contained gaps in the diagnostic fields (Figure 3).

After interactively selecting the 138 unexpected intersections with gaps, another value bar chart showed that DIAG_03 was missing substantially more often than the subsequent diagnosis fields (Figure 5). Entropy calculations showed that all 1776 records in those intersections were for EPITYPE=3 ("birth episode"), all 1776 records were for ADMIMETH=31 ("admitted antepartum"), and 1575 of the records were from one PROCODE.

For peer review only - http://bmjopen.bmj.com/site/about/guidelines.xhtml

[Figure 5 should go approximately here]

Operation code and date fields

We first investigated the operation codes and dates separately, and then together. The code fields exhibited the same characteristics as the diagnosis codes. A combination heatmap showed that most (15,717,333) records exhibited a monotone pattern. However, there were also some gaps (72 set intersections; 2488 records). After we selected those intersections, IGR and entropy calculations showed that 1795 of the records belonged to ADMIMETH=31 and 1588 of the records were from the same PROCODE as the diagnosis gaps.

The operation date fields also exhibited the same characteristics. Most (15,716,187) records exhibited a monotone pattern, but gaps occurred in 89 intersections (3689 records). Again, entropy calculations showed that 2458 of the gaps occurred for ADMIMETH=31 and 2159 of the records were from same PROCODE as above.

Further unexpected patterns were revealed when the operation codes and dates were visualized together, because 45 intersections had no gaps but involved a different number of code and date fields. Each operation code should have a corresponding date in every record (i.e., a block missingness pattern), but 39 out of those 45 intersections contained fewer operation dates than codes, and the other six intersections contained extra dates.

The gaps in the diagnosis codes, and operation codes and dates fields were a surprise to the epidemiologist and to members of NHS Digital's Data Quality team, with whom we subsequently discussed the findings. Of particular concern to NHS Digital were the operation codes, because they affect the process that the NHS Digital Casemix Team use to calculate Healthcare Resource Groups (HRGs; the NHS equivalent of the diagnosis related groups that were pioneered in the USA). HRGs group together clinically similar treatments that involve similar levels of health care resource and, therefore, are one of the building blocks of the NHS's Payment by Results system for hospitals.[48] That system is used because it is much simpler than determining the amount to be paid for each of the tens of thousands of interventions and diagnoses that are used for patients in hospitals.

For peer review only - http://bmjopen.bmj.com/site/about/guidelines.xhtml

DISCUSSION

This study shows how set visualization techniques may be used to investigate and explain patterns of missing data in tabular data. Set visualization was applied by treating all of the records that were missing a given field as a set, so a set intersection comprised the records that were missing the same combination of fields. The two main visualization techniques were bar charts to show each set (i.e., the number of records missing each field) and heatmaps to show set intersections (i.e., the number of records that were only missing each specific combination fields but did have values for the other fields). They were combined with histograms and another bar chart to let the tool scale to the millions of records and thousands of set intersections in the data. The visualizations were essential for enabling users to see and reason about the completely unexpected patterns, and integrating the visualizations with two well-known data mining methods (IGR and entropy) enabled users to pinpoint some of the patterns' origins and explain the underlying structures. The interactive tool we developed is freely available.[39]

Bar charts, heatmaps and histograms are standard visualization techniques, of course. However, in the present study it was the way those techniques and the data mining methods were combined into an easy-to-use interactive tool that was novel, and key to being able to apply set visualization to find and explain the wide variety of patterns of missing data that occurred. To the authors' knowledge, the study is the first time that set visualization has been applied to large-scale EHR data and the study's strengths are: (a) showing the benefits of different set visualization techniques and how they combine to allow a holistic investigation of missing data patterns, and (b) the actionable insights that those visualizations produced for both researchers (the epidemiologist) and organisations (NHS Digital and individual hospitals).

In terms of missing data, most researchers and organisations only count the number of values that are missing in individual fields,[6 7 9 12 15-17] showing the results in either a table or the equivalent of our value bar chart. In our study that bar chart showed the high-level patterns that were dominant (the well-known monotone patterns in the diagnosis codes, operation codes and operation dates), but also and contrary to expectations that a few fields were missing some values instead of being complete.

The most important visualization was the combination heatmap, which we used to visualize set intersections. By using a heatmap, the unexpectedly large number of intersections and the multitude of gaps in the diagnosis codes, operation codes and operation dates all popped out (i.e., were immediately obvious) to users, and closer inspection revealed the discrepancies between pairs of operation codes

For peer review only - http://bmjopen.bmj.com/site/about/guidelines.xhtml

and dates. The tool's interactive data mining then enabled us to pinpoint the origin of some of the unexpected patterns. The few pieces of data quality software that can visualize set intersections are either interactive but only handle small datasets,[36] or generate static plots and necessitate users writing extra computer code to select specific set intersections (e.g., all those with gaps in the operation codes) for further investigation,[35 37 38] rather than interactively selecting those intersections with a few mouse clicks as with our ACE tool. Unlike ACE, none of that other software has any inbuilt data mining functionality.

The combination count and length histograms were useful during the first stage of analysis (Figure 1), to help a user gain a general understanding of the interwoven nature of the patterns of missing data. Had the combination heatmap been used at that stage then the user would have been overwhelmed by the 4371 rows. Our dataset only had 86 fields, but had there been more then the value count histogram would have been similarly useful during the first stage of analysis.

The study had two main limitations. First, the case study only involved one dataset, albeit one from a national organisation that provides similar datasets to many researchers and organisations each year. Second, the tool is primarily designed to work with single data tables. However, longitudinal data would be easy to accommodate by concatenating each dataset and adding a field that stored a date for each dataset. Missing data patterns that occurred throughout the data would be appear as patterns did in the present study, and patterns that were limited to a particular part(s) of the longitudinal data would be revealed by entropy calculations that involved the new field. Missing data patterns in a relational database could be investigated by either analysing each data table separately or joining fields of interest into a new table for analysis. For example, imagine a researcher's database contained information about GP visits, diagnoses and prescriptions. The researcher could process the data to create a new table that contained fields about the visit (Date; Patient ID; GP ID), diagnoses and prescriptions (Drug; Dose). ACE's set intersection heatmap would show that as expected some visits resulted in a prescription whereas others did not, and unexpected patterns such as visits without a date or prescriptions without a dose would pop out as additional rows in the heatmap. The researcher could then use ACE's interactive data mining to determine whether the unexpected patterns were correlated with particular patients, GP practices or diagnoses.

Our approach benefits analysts and researchers by making it easier to check data when it is received. In principle that would have allowed the epidemiologist to remedy the survival time issue by a data

For peer review only - http://bmjopen.bmj.com/site/about/guidelines.xhtml

request amendment, but the time limit for doing that (one month) had passed before the case study started. Regarding the missing dates, as the tool pinpointed the origin as maternity records the epidemiologist made an informed decision to discard the records because she was conducting cardiovascular research. Our approach also benefits data providers by pinpointing where issues occur (e.g., the gaps originated almost entirely in a particular part of a specific hospital). Knowing that, it would be straightforward to provide feedback to rectify the problem via NHS Digital's existing data quality lifecycle[12] and appropriate actions (e.g., fixing IT issues, modifying processes or staff training). Our approach helped communication within NHS Digital, allowing a connection to be made between gaps in the operation code fields and the NHS's Payment by Results system [48] at a national level.

The present study showed how set visualization enables users to identify a variety of unexpected missing data patterns. With further research, that could be developed into a comprehensive library of patterns that users can apply in health data settings to analyse missing data faster and more comprehensively. Further research is also needed to determine the data mining methods that are best suited for determining the structures that explain each type of pattern, extending our use of IGR/entropy and the work of others with decision trees,[2]

CONCLUSIONS

Missing data has widespread impacts on secondary uses of EHRs. Investigations are typically limited to counting the number of values that are missing from key fields, and there is a lack of methods for investigating more complex patterns of missing values, and particularly patterns that are uncommon. A solution is offered by set visualization techniques, which treat records that are missing a given field as a set, and records that are missing multiple fields as set intersections. This study describes how interactive set visualization can be combined with data mining to identify and explain complex patterns of missing values. Using a case study with a 16-million record / 86 field EHR dataset, we demonstrate that set visualization revealed actionable insights that ranged from determining the data were not suitable for analysing short-term survival patterns, to providing feedback to hospitals to improve future data quality, and knock-on consequences for hospital payments.

Acknowledgements

The authors thank the NHS Digital staff who provided guidance during the research and comments about the implications of the findings.

For peer review only - http://bmjopen.bmj.com/site/about/guidelines.xhtml

Funding

This research was supported by the Engineering and Physical Sciences Research Council grant numbers EP/N013980/1 and EP/K503836/1, the British Heart Foundation grant number PG/13/81/30474, and the Alan Turing Institute.

Author contributions

MA and RAR designed the software. MA developed the software. MSH provided subject matter expertise about HES data. RAR wrote the first full version of the manuscript. All authors contributed to the case study and approved the final manuscript.

Competing interests

None.

Patient consent for publication

Not required.

Ethics approval

The study was approved by the University of Leeds MaPS and Engineering joint Faculty Research Ethics Committee (LIDA 15-002).

Data availability statement

The dataset was provided by NHS Digital (request number DARS-NIC-17649-G0X4B-v0.6) and, due to data governance restrictions, cannot be made openly available.

Provenance and peer review

Not commissioned; externally peer reviewed.

For peer review only

Figure captions

Figure 1 A workflow for investigating the patterns of missing values with ACE. The two levels of iteration are to: (a) select subsets of fields, (b) select groups of set intersections to explain a pattern in terms of the values of other fields.

Figure 2 A value bar chart showing the sets (the number of missing values in each field; NB: the Y-axis uses a log scale).

Figure 3 ACE utilises a tab pane layout with color-coded tabs (a). Each tab is split into a visualization panel (b) and a control panel (c), and the details depend on the type of visualization. This figure shows a heatmap of expected (d) and unexpected (e) set intersections that involve the DIAG_nn fields. A context menu (f) highlights various actions that can be performed on a selected intersection. The control panel contains a dataset summary (g), visualization size (h), heatmap legend (i), sorting methods (j), and filtering options (k).

Figure 4 A combination length histogram, which shows that most of the set intersections involved 23 – 64 fields.

Figure 5 A value bar chart showing the number of records in each set for the whole dataset (left) and the set intersections that, unexpectedly, contained gaps in the DIAG_nn fields (right).

REFERENCES

1. Khare R, Utidjian L, Ruth BJ, et al. A longitudinal analysis of data quality in a large pediatric data research network. *J Am Med Inform Assoc* 2017;24(6):1072-79.
2. Tierney NJ, Harden FA, Harden MJ, et al. Using decision trees to understand structure in missing data. *BMJ Open* 2015;5(6):e007450.
3. Collins GS, Reitsma JB, Altman DG, et al. Transparent reporting of a multivariable prediction model for individual prognosis or diagnosis (TRIPOD): the TRIPOD statement. *Journal of British Surgery* 2015;102(3):148-58.
4. Li R, Chen Y, Moore JH. Integration of genetic and clinical information to improve imputation of data missing from electronic health records. *J Am Med Inform Assoc* 2019;26(10):1056-63.

For peer review only - http://bmjopen.bmj.com/site/about/guidelines.xhtml

5. Kharrazi H, Wang C, Scharfstein D. Prospective EHR-based clinical trials: the challenge of missing data. *J Gen Intern Med* 2014;29:976-78.
6. Mathur R, Bhaskaran K, Chaturvedi N, et al. Completeness and usability of ethnicity data in UK-based primary care and hospital databases. *Journal of Public Health* 2014;36(4):684-92.
7. Murray J, Saxena S, Modi N, et al. Quality of routine hospital birth records and the feasibility of their use for creating birth cohorts. *Journal of Public Health* 2013;35(2):298-307.
8. Knight HE, Gurol-Urganci I, Mahmood TA, et al. Evaluating maternity care using national administrative health datasets: How are statistics affected by the quality of data on method of delivery? *BMC Health Services Research* 2013;13(1):200.
9. Smith M, Lix LM, Azimaee M, et al. Assessing the quality of administrative data for research: a framework from the Manitoba Centre for Health Policy. *J Am Med Inform Assoc* 2018;25(3):224-29.
10. The processing cycle and HES data quality: NHS Digital; 2022 [Available from: https://digital.nhs.uk/data-and-information/data-tools-and-services/data-services/hospital-episode-statistics/the-processing-cycle-and-hes-data-quality# accessed 13 May 2022.
11. Farquhar M. AHRQ quality indicators. Patient safety and quality: An evidence-based handbook for nurses: Agency for Healthcare Research and Quality (US) 2008.
12. HES data dictionary: Admitted patient care: NHS Digital; 2017 [Available from: http://content.digital.nhs.uk/media/25188/DD-APC-V10/pdf/DD-APC-V10.pdf accessed 13 May 2022.
13. Hospital Episode Statistics (HES): NHS Digital; 2019 [Available from: https://digital.nhs.uk/data-and-information/data-tools-and-services/data-services/hospital-episode-statistics accessed 13 May 2022.
14. Users, uses and access to Hospital Episode Statistics: NHS Digital; 2020 [Available from: https://digital.nhs.uk/data-and-information/data-tools-and-services/data-services/hospital-episode-statistics/users-uses-and-access-to-hospital-episode-statistics accessed 13 May 2022.
15. Goldstein BA, Navar AM, Pencina MJ, et al. Opportunities and challenges in developing risk prediction models with electronic health records data: a systematic review. *J Am Med Inform Assoc* 2017;24(1):198-208.
16. Herbert A, Wijlaars L, Zylbersztejn A, et al. Data resource profile: Hospital episode statistics admitted patient care (HES APC). *Int J Epidemiol* 2017;46(4):1093-93i.
17. Pringle M, Ward P, Chilvers C. Assessment of the completeness and accuracy of computer medical records in four practices committed to recording data on computer. *Br J Gen Pract* 1995;45(399):537-41.
18. Boslett AJ, Denham A, Hill EL, et al. Unclassified drug overdose deaths in the opioid crisis: emerging patterns of inequity. *J Am Med Inform Assoc* 2019;26(8-9):767-77.
19. Rubin DB. Characterizing the estimation of parameters in incomplete-data problems. *Journal of the American Statistical Association* 1974;69(346):467-74.
20. Enders KE. Applied missing data analysis. New York: Guilford 2010.
21. Monroe M, Lan R, Lee H, et al. Temporal event sequence simplification. *IEEE Trans Vis Comput Graphics* 2013;19:2227-36.
22. Caban JJ, Gotz D. Visual analytics in healthcare–opportunities and research challenges. *J Am Med Inform Assoc* 2015;22:260-62.
23. Wu DT, Chen AT, Manning JD, et al. Evaluating visual analytics for health informatics applications: a systematic review from the American Medical Informatics Association Visual Analytics Working Group Task Force on Evaluation. *J Am Med Inform Assoc* 2019;26(4):314-23.
24. Rind A, Wang TD, Wolfgang A, et al. Interactive information visualization to explore and query electronic health records. *Foundations and Trends in Human-Computer Interaction* 2013;5:207-98.

For peer review only - http://bmjopen.bmj.com/site/about/guidelines.xhtml

25. West VL, Borland D, Hammond WE. Innovative information visualization of electronic health record data: a systematic review. *J Am Med Inform Assoc* 2015;22:330-39.
26. Dowding D, Randell R, Gardner P, et al. Dashboards for improving patient care: Review of the literature. *Int J Med Inf* 2015;84(2):87-100.
27. Alper B, Riche N, Ramos G, et al. Design study of LineSets, a novel set visualization technique. *IEEE Trans Vis Comput Graphics* 2011;17(12):2259-67.
28. Dörk M, Riche NH, Ramos G, et al. Pivotpaths: Strolling through faceted information spaces. *IEEE Trans Vis Comput Graphics* 2012;18(12):2709-18.
29. Freiler W, Matkovic K, Hauser H. Interactive visual analysis of set-typed data. *IEEE Trans Vis Comput Graphics* 2008;14(6):1340-47.
30. Noselli M, Mason D, Mohammed MA, et al. MonAT: A Visual Web-based Tool to Profile Health Data Quality. HEALTHINF2017:26-34.
31. Yalcin MA, Elmqvist N, Bederson BB. AggreSet: Rich and scalable set exploration using visualizations of element aggregations. *IEEE Trans Vis Comput Graphics* 2015;22(1):688-97.
32. OpenRefine: A free, open source, powerful tool for working with messy data [program], 2018. Available from: http://openrefine.org/ accessed 13 May 2022.
33. Kandel S, Parikh R, Paepcke A, et al. Profiler: integrated statistical analysis and visualization for data quality assessment. Proceedings of the International Working Conference on Advanced Visual Interfaces: ACM 2012:547-54.
34. Trifacta Wrangler [program], 2020. Available from: https://www.trifacta.com/ accessed 13 May 2022.
35. Templ M, Alfons A, Filzmoser P. Exploring incomplete data using visualization techniques. *Advances in Data Analysis and Classification* 2012;6(1):29-47.
36. Lex A, Gehlenborg N, Strobelt H, et al. UpSet: visualization of intersecting sets. *IEEE Trans Vis Comput Graphics* 2014;20(12):1983-92.
37. Conway JR, Lex A, Gehlenborg N. UpSetR: an R package for the visualization of intersecting sets and their properties. *Bioinformatics* 2017;33(18):2938-40.
38. UpSetPlot [program], 2021. Available from: https://pypi.org/project/UpSetPlot/ accessed 13 May 2022.
39. The ACE software, and training materials for visualizing missing data and set-type data [program], 2022. Available from: https://doi.org/10.5518/1133 accessed 13 May 2022.
40. HyperSQL Homepage [program]. 2.5.0 version, 2019. Available from: http://hsqldb.org/ accessed 13 May 2022.
41. Arbesser C, Spechtenhauser F, Mühlbacher T, et al. Visplause: Visual data quality assessment of many time series using plausibility checks. *IEEE Trans Vis Comput Graphics* 2016;23(1):641-50.
42. Tierney NJ, Cook DH. Expanding tidy data principles to facilitate missing data exploration, visualization and assessment of imputations. *arXiv preprint arXiv:180902264* 2018
43. Menon S, Zaidi E. Market guide for data preparation tools 2019 [Available from: https://www.gartner.com/en/documents/3906957/market-guide-for-data-preparation-tools accessed 13 May 2022.
44. Honaker J, King G, Blackwell M. Amelia II: A program for missing data. *Journal of Statistical Software* 2011;45:1-47.
45. Matrix reordering methods for table and network visualization. Computer Graphics Forum; 2016. Wiley Online Library.
46. Quinlan JR. Induction of decision trees. *Machine Learning* 1986;1(1):81-106.
47. Shannon CE. A mathematical theory of communication. *ACM SIGMOBILE Mobile Computing and Communications Review* 2001;5(1):3-55.
48. A simple guide to payment by results: Department of Health; 2012 [Available from: https://assets.publishing.service.gov.uk/government/uploads/system/uploads/attachment_data/file/213150/PbR-Simple-Guide-FINAL.pdf accessed 13 May 2022.

For peer review only - http://bmjopen.bmj.com/site/about/guidelines.xhtml

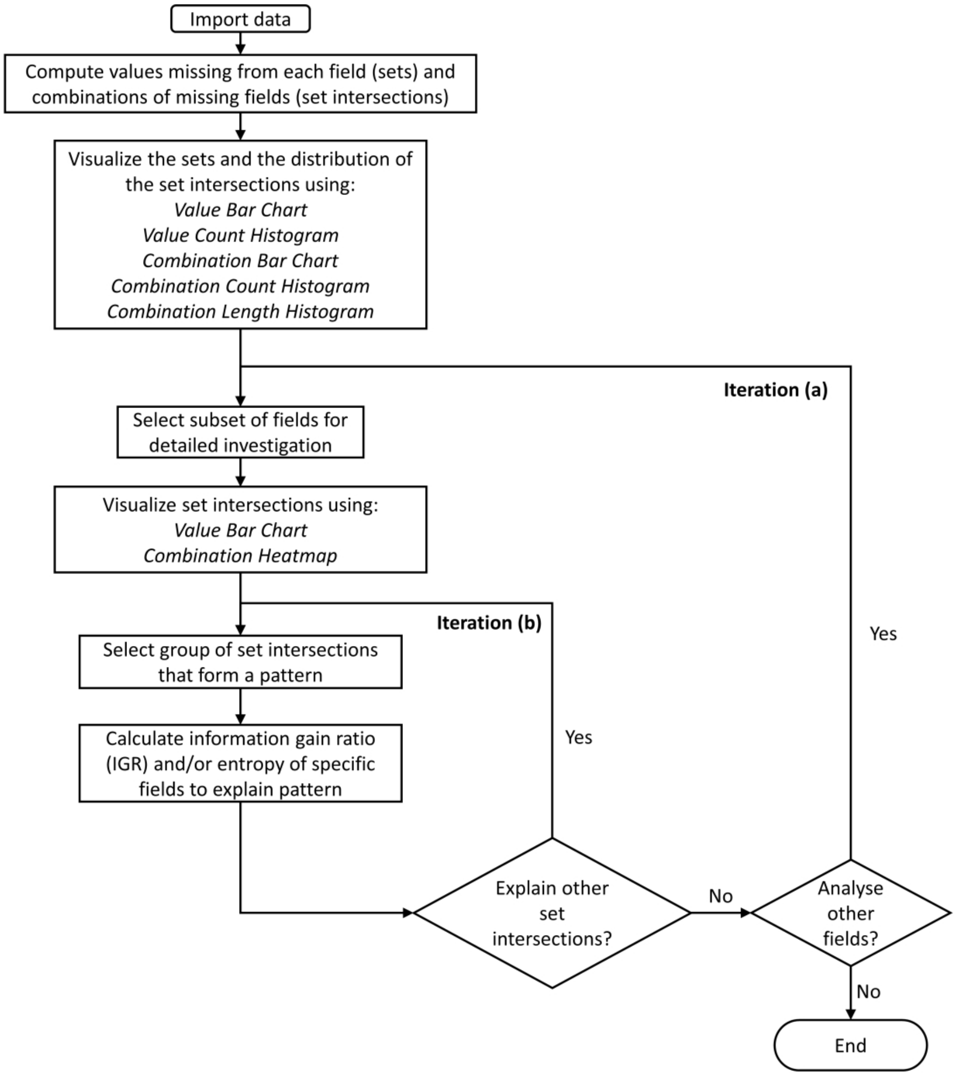

Figure 1 A workflow for investigating the patterns of missing values with ACE. The two levels of iteration are to: (a) select subsets of fields, (b) select groups of set intersections to explain a pattern in terms of the values of other fields.

1000x1119mm (96 x 96 DPI)

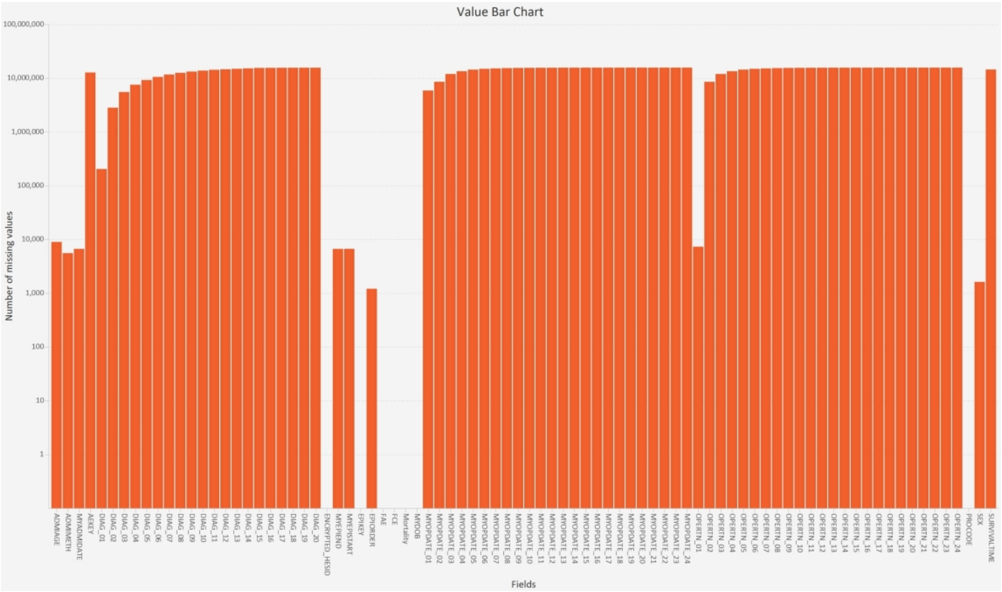

Figure 2 A value bar chart showing the sets (the number of missing values in each field; NB: the Y-axis uses a log scale).

567x333mm (144 x 144 DPI)

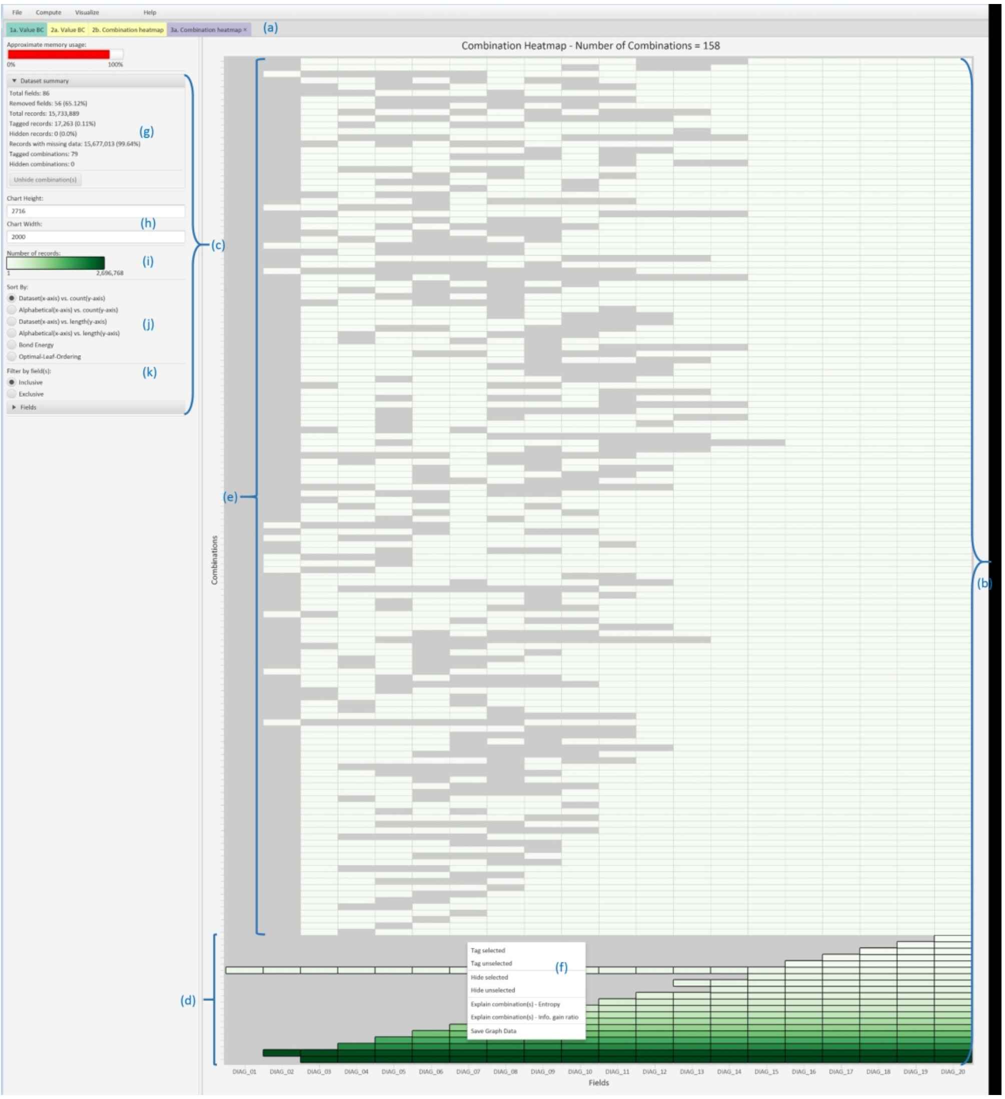

Figure 3 ACE utilises a tab pane layout with color-coded tabs (a). Each tab is split into a visualization panel (b) and a control panel (c), and the details depend on the type of visualization. This figure shows a heatmap of expected (d) and unexpected (e) set intersections that involve the DIAG_nn fields. A context menu (f) highlights various actions that can be performed on a selected intersection. The control panel contains a dataset summary (g), visualization size (h), heatmap legend (i), sorting methods (j), and filtering options (k).

1278x1392mm (59 x 59 DPI)

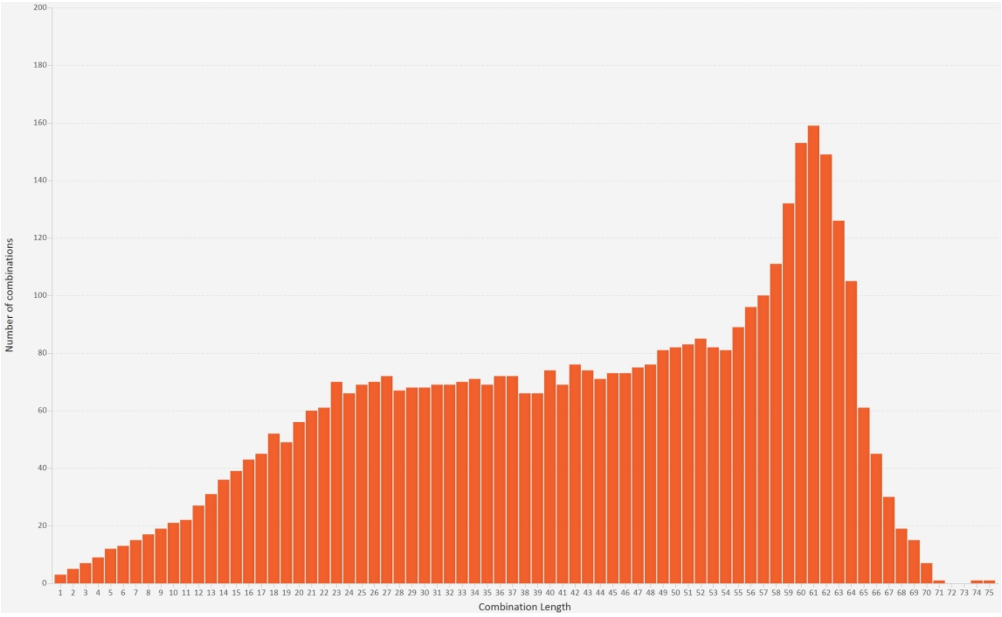

Figure 4 A combination length histogram, which shows that most of the set intersections involved 23 – 64 fields.

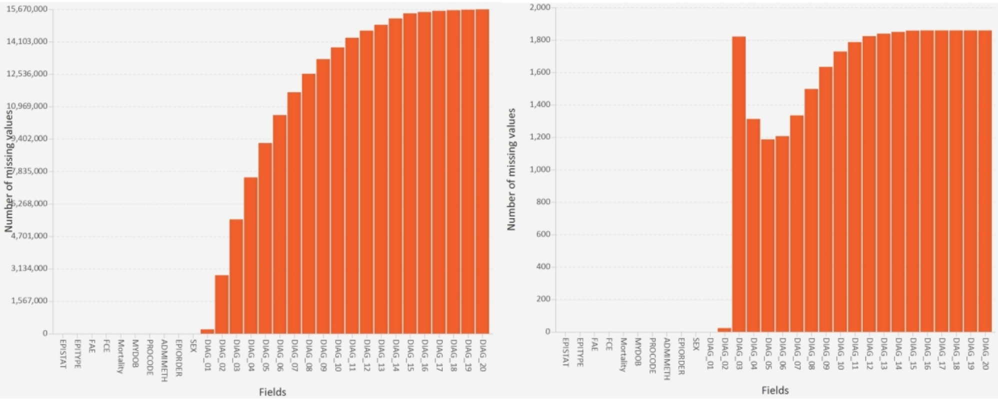

Figure 5 A value bar chart showing the number of records in each set for the whole dataset (left) and the set intersections that, unexpectedly, contained gaps in the DIAG_nn fields (right).

507x200mm (144 x 144 DPI)

Reporting checklist for prediction model development/validation.

Based on the TRIPOD guidelines.

Instructions to authors

Complete this checklist by entering the page numbers from your manuscript where readers will find each of the items listed below.

Your article may not currently address all the items on the checklist. Please modify your text to include the missing information. If you are certain that an item does not apply, please write "n/a" and provide a short explanation.

Upload your completed checklist as an extra file when you submit to a journal.

In your methods section, say that you used the TRIPODreporting guidelines, and cite them as:

Collins GS, Reitsma JB, Altman DG, Moons KG. Transparent reporting of a multivariable prediction model for individual prognosis or diagnosis (TRIPOD): The TRIPOD statement.

A note to the reviewers: We have used the TRIPOD reporting guidelines, because that is better-suited to this type of research than other guidelines. However, it should be noted that TRIPOD is designed for prediction model studies rather than those that develop visualization software, which is why we have marked a number of the guidelines as n/a (particularly those that are specifically about prediction itself or the statistical analysis methods). For some guidelines we have added comments as well as the page number, to help the reader.

Reporting Item	Page Number

For peer review only

**Title**			
	#1	Identify the study as developing and / or validating a multivariable prediction model, the target population, and the outcome to be predicted.	1 (NB: the study developed a visualization tool, rather than a statistical model)
**Abstract**			
	#2	Provide a summary of objectives, study design, setting, participants, sample size, predictors, outcome, statistical analysis, results, and conclusions.	3
**Introduction**			
	#3a	Explain the medical context (including whether diagnostic or prognostic) and rationale for developing or validating the multivariable prediction model, including references to existing models.	4 (and p5/6 for references to existing tools)
	#3b	Specify the objectives, including whether the study describes the development or validation of the model or both.	4
**Methods**			
Source of data	#4a	Describe the study design or source of data (e.g., randomized trial, cohort, or registry data), separately for the	6

For peer review only

		development and validation data sets, if applicable.	
Source of data	#4b	Specify the key study dates, including start of accrual; end of accrual; and, if applicable, end of follow-up.	n/a
Participants	#5a	Specify key elements of the study setting (e.g., primary care, secondary care, general population) including number and location of centres.	6
Participants	#5b	Describe eligibility criteria for participants.	6
Participants	#5c	Give details of treatments received, if relevant	n/a
Outcome	#6a	Clearly define the outcome that is predicted by the prediction model, including how and when assessed.	n/a
Outcome	#6b	Report any actions to blind assessment of the outcome to be predicted.	n/a
Predictors	#7a	Clearly define all predictors used in developing or validating the multivariable prediction model, including how and when they were measured	n/a

For peer review only

Predictors	#7b	Report any actions to blind assessment of predictors for the outcome and other predictors.	n/a
Sample size	#8	Explain how the study size was arrived at.	6 (to demonstrate scalability to large datasets, the study used a whole year of NHS APC data, apart from the exclusion criteria described on p6)
Missing data	#9	Describe how missing data were handled (e.g., complete-case analysis, single imputation, multiple imputation) with details of any imputation method.	7-8 (described in the tool's design)
Statistical analysis methods	#10a	If you are developing a prediction model describe how predictors were handled in the analyses.	n/a
Statistical analysis methods	#10b	If you are developing a prediction model, specify type of model, all model-building procedures (including any predictor selection), and method for internal validation.	n/a
Statistical analysis methods	#10c	If you are validating a prediction model, describe how the predictions were calculated.	n/a

For peer review only

Statistical analysis methods	#10d	Specify all measures used to assess model performance and, if relevant, to compare multiple models.	n/a
Statistical analysis methods	#10e	If you are validating a prediction model, describe any model updating (e.g., recalibration) arising from the validation, if done	n/a
Risk groups	#11	Provide details on how risk groups were created, if done.	n/a
Development vs. validation	#12	For validation, identify any differences from the development data in setting, eligibility criteria, outcome, and predictors.	n/a
**Results**			
Participants	#13a	Describe the flow of participants through the study, including the number of participants with and without the outcome and, if applicable, a summary of the follow-up time. A diagram may be helpful.	9 (the analysis workflow)
Participants	#13b	Describe the characteristics of the participants (basic demographics, clinical features, available predictors), including	9 (the quantity of missing values)

For peer review only

		the number of participants with missing data for predictors and outcome.	
Participants	#13c	For validation, show a comparison with the development data of the distribution of important variables (demographics, predictors and outcome).	n/a
Model development	#14a	If developing a model, specify the number of participants and outcome events in each analysis.	9-11 (the Results is divided into three sections, which each describe the outcome for that part of the analysis)
Model development	#14b	If developing a model, report the unadjusted association, if calculated between each candidate predictor and outcome.	n/a
Model specification	#15a	If developing a model, present the full prediction model to allow predictions for individuals (i.e., all regression coefficients, and model intercept or baseline survival at a given time point).	n/a
Model specification	#15b	If developing a prediction model, explain how to the use it.	n/a
Model performance	#16	Report performance measures (with CIs) for the prediction model.	n/a

For peer review only

Model-updating	#17	If validating a model, report the results from any model updating, if done (i.e., model specification, model performance).	n/a
**Discussion**			
Limitations	#18	Discuss any limitations of the study (such as nonrepresentative sample, few events per predictor, missing data).	13
Interpretation	#19a	For validation, discuss the results with reference to performance in the development data, and any other validation data	n/a
Interpretation	#19b	Give an overall interpretation of the results, considering objectives, limitations, results from similar studies, and other relevant evidence.	12
Implications	#20	Discuss the potential clinical use of the model and implications for future research	13
**Other information**			
Supplementary information	#21	Provide information about the availability of supplementary resources, such as	12 (the tool is freely available; DOI in the references)

For peer review only

		study protocol, Web calculator, and data sets.	
Funding	#22	Give the source of funding and the role of the funders for the present study.	14

None The TRIPOD checklist is distributed under the terms of the Creative Commons Attribution License CC-BY. This checklist can be completed online using https://www.goodreports.org/, a tool made by the EQUATOR Network in collaboration with Penelope.ai

For peer review only
